# Beyond syndromic management: Opportunities for diagnosis-based treatment of sexually transmitted infections in low- and middle-income countries

**DOI:** 10.1371/journal.pone.0196209

**Published:** 2018-04-24

**Authors:** Nigel J. Garrett, Farzana Osman, Bhavna Maharaj, Nivashnee Naicker, Andrew Gibbs, Emily Norman, Natasha Samsunder, Hope Ngobese, Nireshni Mitchev, Ravesh Singh, Salim S. Abdool Karim, Ayesha B. M. Kharsany, Koleka Mlisana, Anne Rompalo, Adrian Mindel

**Affiliations:** 1 Centre for the AIDS Programme of Research in South Africa (CAPRISA), Durban, South Africa; 2 School of Nursing and Public Health, Discipline of Public Health Medicine, University of KwaZulu-Natal, Durban, South Africa; 3 South African Medical Research Council, Durban, South Africa; 4 Department of Epidemiology, Columbia University, New York City, United States of America; 5 Prince Cyril Zulu Communicable Disease Centre, eThekwini Municipality, Durban, South Africa; 6 Department of Microbiology, University of KwaZulu-Natal, Durban, South Africa; 7 National Health Laboratory Service, Durban, South Africa; 8 Johns Hopkins University, Baltimore, United States of America; University of Ottawa, CANADA

## Abstract

**Introduction:**

In light of the limited impact the syndromic management approach has had on the global sexually transmitted infection (STI) epidemic, we assessed a care model comprising point-of-care (POC) STI testing, immediate treatment, and expedited partner therapy (EPT) among a cohort of young women at high HIV risk in South Africa.

**Methods and findings:**

HIV negative women presenting for STI care underwent POC testing for *Chlamydia trachomatis* (CT), *Neisseria gonorrhoeae* (NG) and *Trichomonas vaginalis* (TV), and swabs were sent for NG culture and susceptibility testing. Results were available within 2 hours and women with STIs were immediately treated and offered EPT packs, including medication, condoms, and information for sexual partners. An EPT questionnaire was administered after one week, and women retested for STIs after 6 and 12 weeks. 267 women, median age 23 (IQR 21–26), were recruited and 88.4% (236/267) reported genital symptoms. STI prevalence was CT 18.4% (95%CI 13.7–23.0), NG 5.2% (95%CI 2.6–7.9) and TV 3.0% (95%CI 1.0–5.0). After 12 weeks, all but one NG and two CT infections were cleared. No cephalosporin-resistant NG was detected. Of 63/267 women (23.6%) diagnosed with STIs, 98.4% (62/63) were offered and 87.1% (54/62) accepted EPT. At one week 88.9% (48/54) stated that their partner had taken the medication. No allergic reactions or social harms were reported. Of 51 women completing 6-week follow up, detection rates were lower amongst women receiving EPT (2.2%, 1/46) compared to those who did not (40.0%, 2/5), p = 0.023. During focus group discussions women supported the care model, because they received a rapid, specific diagnosis, and could facilitate their partners’ treatment.

**Conclusions:**

POC STI testing and EPT were acceptable to young South African women and their partners, and could play an important role in reducing STI reinfection rates and HIV risk. Larger studies should evaluate the feasibility and cost-effectiveness of implementing this strategy at population level.

## Introduction

More than three decades into the HIV epidemic, little progress has been made in preventing HIV acquisitions among young women in Sub-Saharan Africa [1]. Other sexually transmitted infections (STIs) have long been recognized as major modifiable risk factors for HIV infection [2,3,4,5,6], but effective STI care still does not reach the most vulnerable, especially in low- and middle-income countries (LMICs) with high HIV burden. In addition, there are several major complications of STIs including pelvic inflammatory disease with resultant tubal infertility, risk of ectopic pregnancy and chronic pelvic pain; cervical and other genital tract tumours, spread to the new born baby and psychosexual problems as well as a substantial economic burden [7,8,9].

Despite the major health burden of STIs, governments in many LMICs have persisted with the model of syndromic management, mainly because of limited resources, the lack of adequate laboratory services, and to deliver immediate care by nurses [10,11,12]. The limitations of this approach have been well documented and include the inability to detect asymptomatic STIs [12,13,14], the poor positive predictive value of syndromic treatment resulting in the overuse of antibiotics (of particular concern in relation to antibiotic resistance to gonorrhoea) [15], the lack of antimicrobial susceptibility testing, and limited opportunities for routine surveillance. In South Africa, syndromic management was implemented in 1995, and the prevalence of STIs remains unchanged [12,16,17]. Innovative, low-cost diagnostic models for enhanced STI care are urgently required.

Another reason for poor outcomes with syndromic management is that there are limited opportunities to find and treat sexual partners with appropriate medication. Diagnostic services would allow for a specific diagnosis and appropriate treatment for the index case as well as their partners. Several studies have shown that expedited partner therapy (EPT) for bacterial STIs delivered by the index case or through pharmacies can reduce reinfection rates among women [18,19]. While EPT has been implemented in some settings in the United States, only a few studies from LMICs have suggested that EPT may be effective at increasing partner treatment and reducing reinfection rates [20,21]. In South Africa, partner-delivered patient referral remains the mainstay of contact tracing, but has been largely unsuccessful with partner treatment rates below 22% [22].

The lack of data on the acceptability, feasibility and effectiveness of EPT in LMICs with high HIV incidence settings is a major gap. If effective, this intervention could contribute to the reduction of high STI rates among young women, by increasing partner treatment, thereby lowering reinfection rates, and by potentially addressing other contributing factors including stigma and the lack of knowledge about STIs [20]. This in turn could lead to a reduction in HIV acquisition. The current interest in more affordable STI diagnostics, including point-of-care (POC) assays, could accelerate the introduction of EPT, as countries consider a shift from syndromic to diagnostic management.

The objective of this study was to evaluate a POC diagnostic STI model combined with an EPT intervention among young women at high HIV risk in South Africa.

## Methods

### Study design

This prospective cohort pilot study (CAPRISA 083) evaluated an enhanced STI management intervention including POC STI testing, immediate supervised treatment, and EPT among young women in South Africa. Women who consented were screened for STIs and bacterial vaginosis (BV). Those with a STI and/or BV were treated and then booked to retest at the clinic after 6 and 12 weeks.

### Study setting

The CAPRISA 083 study was conducted in the Prince Cyril Zulu Communicable Disease Centre (PCZCDC), a large public healthcare clinic in the city centre of Durban, KwaZulu-Natal, South Africa between May 2016 and January 2017. KwaZulu-Natal has one of the highest HIV incidence rates among young women globally (up to 9%) [23]. The clinic provides general primary health care services for adults free of charge, focuses on the diagnosis and treatment of HIV and Tuberculosis, and offers syndromic management for STIs in line with the South African Department of Health guidelines [24].

### Study population

Women attending the clinic for STI care were recruited and, if willing to participate, provided written informed consent for the diagnostic care intervention instead of standard syndromic management. Eligible patients were female, age 18–40 years, who had an HIV negative antibody test. As this was a proof-of-concept study, HIV positive or pregnant women, and sex workers were excluded, because they either required specialized care, or were not the ideal target population for the EPT intervention due to multiple unknown clients. Ethical approval was granted by the Biomedical Research Ethics Committee of the University of KwaZulu-Natal (BFC410/15), and the eThekwini Municipality gave permission to conduct the study at the PCZCDC. Considering participants were offered a diagnostic intervention outside standard care in South Africa, the study was registered online at www.clinicaltrials.gov under study number NCT03407586.

### Clinical assessment and sample collection

In order to streamline study visits, once a participant was enrolled, a professional nurse initially collected two blind vaginal swabs for STI testing and microscopy. While the testing was underway in the clinic laboratory, the nurse continued with study procedures. First, she administered a structured sexual behavioural questionnaire, which included questions on the reasons for attending the clinic, recent sexual activity, number of partners, condom and contraceptive use, and pregnancy desire. The nurse then performed a general physical examination, followed by a pelvic examination with a speculum to assess for vulval, vaginal and cervical abnormalities and to take additional study specimens including an Eswab™ to enable bacterial culture and antimicrobial susceptibility testing.

### POC STI and antimicrobial susceptibility testing

All POC STI tests were conducted by laboratory technologists with experience with the GeneXpert® platform at the onsite laboratory and were processed within approximately 2 hours. POC tests included the Xpert® CT/NG assay (Cepheid, Sunnydale, California, US) to test for *Chlamydia trachomatis* (CT) and *Neisseria gonorrhoeae* (NG), the OSOM® Rapid Trichomonas Test (Sekisui Diagnostics, Lexington, MA, US) to detect *Trichomonas vaginalis* (TV), and Gram stain microscopy to assess for candidiasis and BV by Nugent Score. Any cases of NG were further investigated for culture and antimicrobial susceptibility at the Inkosi Albert Luthuli National Health Laboratory Services reference laboratory using the collected Eswab™.

### Immediate single dose treatment

Women diagnosed with CT, NG or TV were offered immediate supervised treatment with single doses of antibiotics on the same visit. STI treatment was organism-specific following international guidelines, and was also compatible with national guidelines: Ceftriaxone 250mg intramuscular and Azithromycin 1g oral for NG, Azithromycin 1g oral for CT, and Metronidazole 2g oral for TV [24,25]. Women diagnosed with intermediate flora or BV (Nugent Scores 4–6 and 7–10, respectively) were offered a single dose of oral Metronidazole 2g, and those with candidiasis were treated with one Clotrimazole pessary and Clotrimazole cream, if vulval symptoms were present.

### Expedited partner therapy

Once treated for NG, CT or TV, women were offered EPT packs, which included appropriate medication, male condoms and an information leaflet (S1 Fig) for the current partner/s. Partner medication was equivalent to that received by the index case, except that NG was treated with a single dose of oral Cefixime 400mg, instead of single dose intramuscular Ceftriaxone 250mg. The information leaflet was available in English or isiZulu, the local language, and contained information on STIs, medication administration, potential side effects, and a contact phone number, that the partner could call, if required. One week after their visit, women were contacted by phone to administer an EPT questionnaire, and were then retested for STIs in the clinic after 6 and 12 weeks.

### Focus group discussions on POC testing and EPT

Women diagnosed with a STI were invited to take part in focus group discussions to learn more about their experiences with the POC STI testing model and the feasibility and acceptability of EPT. Participants provided separate written informed consent and then joined one of six sessions attended by 4 to 6 participants, which were conducted by one trained female nurse psychologist and a female assistant. Women were encouraged to share their experiences with disclosing the STI diagnosis to their partners and handing over the EPT pack. The nurse then specifically enquired about potential social harms, including intimate partner violence.

### Data analysis

Study data were collected and managed using REDCap electronic data capture tools (Vanderbilt University, Nashville, TN, US), checked for internal validity and analyzed using SAS version 9.4 (SAS Institute Inc., Cary, NC, USA). Baseline characteristics of the study participants were summarized using descriptive statistics expressed as means with standard deviation or medians with interquartile ranges (IQR) for continuous variables and proportions for categorical variables. An independent samples t-test was used to compare two means of continuous variables, while the Wilcoxon-Mann-Whitney test was used to compare two medians. Proportions were compared using Fisher’s exact test. Confidence intervals were calculated using the Wald interval. A generalized estimating equation (GEE) model using a binomial distribution and accounting for repeated measures was used to determine the effect of time on each STI. All focus group discussions were digitally recorded, transcribed and translated into English, and data were analysed thematically, using an open coding structure.

## Results

### Characteristics of study population

A total of 267 HIV negative women, median age 23 years (IQR 21–26), were enrolled into the study. HIV positive women (screening prevalence 39.1%) and pregnant women (9.1%) were excluded. Almost all women (99.6%, 262/263) had completed at least some secondary education, and most (88.4%, 236/267) presented to the clinic because of STI symptoms (Table 1). All women were sexually active and the majority reported only one male partner during the past 2 months, with the younger women (<25 year olds vs ≥25 year olds) having slightly more sexual partners in the past 12 months (p = 0.023).

**Table 1:** Characteristics of 267 young women attending STI care.

Variable	Category	Overall (N = 267) % (n/N)	< 25 years (N = 177) % (n/N)	≥ 25 years (N = 90) % (n/N)	p-value
Age	Median (IQR)	23 (21–26)			
Highest level of education	Primary	0.4 (1/263)	0	1.1 (1/89)	0.457
	Secondary	73.0 (192/263)	73.6 (128/174)	71.9 (64/89)	
	Tertiary	26.6 (70/263)	26.4 (46/174)	27.0 (24/89)	
Reason for presentation	STI Symptoms	88.4 (236/267)	88.1 (156/177)	88.9 (80/90)	0.976
	Asymptomatic check-up	7.9 (21/267)	7.9 (14/177)	7.8 (7/90)	
	Partner contact slip	0.7 (2/267)	1.1 (2/177)	0	
	Other^a^	3.0 (8/267)	2.8 (5/177)	3.3 (3/90)	
Number of sex partners past 2 months	Median (IQR)	1 (1–1)	1 (1–1)	1 (1–1)	0.257
Number of sex partners past 12 months	Median (IQR)	1 (1–2)	1 (1–2)	1 (1–1)	0.023
Gender of sex partner/s	Men	98.5 (263/267)	97.7 (173/177)	100 (90/90)	0.304
	Men and women	1.5 (4/267)	2.3 (4/177)	0	
Type of sex^b^	Vaginal	100 (267/267)	100 (177/177)	100 (90/90)	-
	Oral	28.5 (76/267)	29.4 (52/177)	26.7 (24/90)	0.670
	Anal	4.9 (13/267)	4.5 (8/177)	5.6 (5/90)	0.767
Any condom use with partner(s)	Yes	68.2 (182/267)	73.4 (130/177)	57.8 (52/90)	0.012
Frequency of condom use	Always	3.7 (10/267)	2.8 (5/177)	5.6 (5/90)	0.011
	Sometimes	64.4 (172/267)	70.6 (125/177)	52.2 (47/90)	
	Never	31.8 (85/267)	26.6 (47/177)	42.2 (38/90)	
Contraception use	Yes	34.1 (91/267)	29.9 (53/177)	42.2 (38/90)	0.056
	Progesterone injections^c^	59.3 (54/91)	58.5 (31/53)	60.5 (23/38)	0.913
	Subdermal Implant^d^	19.8 (18/91)	22.6 (12/53)	15.8 (6/38)	
	Oral contraceptive pills	11.0 (10/91)	9.4 (5/53)	13.2 (5/38)	
	Condom only	7.7 (7/91)	7.5 (4/53)	7.9 (3/38)	
	Intra-uterine device	2.2 (2/91)	1.9 (1/53)	2.6 (1/38)	
Currently trying to conceive a child	Yes	10.5 (28/267)	6.8 (12/177)	17.8 (16/90)	0.010
Concerned about unplanned pregnancy	Yes	68.2 (182/267)	75.7 (134/177)	53.3 (48/90)	<0.001
Genital examination	Abnormal	46.4 (124/267)	47.5 (84/177)	44.4 (40/90)	0.698

^a^ 5 participants specified the ‘other’ reason for presenting at the clinic, i.e. flu, headache/dizziness, chest pain, contraception and tonsillitis.

^b^ Percentages add up to >100% because participants could choose more than one answer.

^c^ Progesterone injections were either medroxyprogesterone acetate or norethisterone enantate injections.

^d^ Etonogestrel Implanon or Nexplanon implants

Overall, 68.2% (182/267) of women stated that they used condoms with their partners, although only 3.7% (10/267) used them consistently. One third of women (34.1%, 91/267) used contraception (29.9% of <25 year olds), while two thirds (68.2%, 182/267), and especially the younger women (75.7% vs 53.3%, p = <0.001), were concerned about an unplanned pregnancy. Almost half the women (46.8%, 124/267) had at least one abnormality on genital examination, including 84.7% (105/124) with vaginal discharge, 21.0% (26/124) with vulval signs (warts, vulvitis, rash or vesicles) and 9.7% (12/124) with cervical abnormalities (warts, discharge or inflammation).

### STI and BV prevalence and antimicrobial susceptibility

Of the 267 women, 23.6% [95% Confidence interval (CI) 18.5–28.7%] were diagnosed with at least one of the three STIs (Table 2). The overall prevalence of CT was 18.4% (95%CI 13.7–23.0), and was 20.3% (95%CI 14.4–26.3) among women less than 25 years old. The prevalence of NG was 5.2% (95%CI 2.6–7.9) and of TV 3.0% (95%CI 1.0–5.0). Seven women were diagnosed with both CT and NG, and one woman had both CT and TV infections. Two thirds of women (69.3%, 185/267) had evidence of abnormal vaginal flora (33.7% BV and 35.6% intermediate flora) based on Nugent Score, and 18.0% (48/267) were diagnosed with candidiasis. A total of 19.5% (52/267) reported symptoms, but had no STI, abnormal flora or candidiasis on laboratory examination. Of the 14 women diagnosed with NG on Xpert® CT/NG, 64.3% (9/14) grew NG on culture. 77.7% (7/9) were resistant to penicillin and 44.4% (4/9) to ciprofloxacin, but no cephalosporin resistance was identified.

**Table 2:** Prevalence of STIs, bacterial vaginosis and candidiasis at baseline in 267 young South African women.

Variable		Overall (N = 267) % (95% Cl)	< 25 years (N = 177) % (95% CI)	>25 years (N = 90) % (95% CI)	p-value
*C*. *trachomatis* (CT)		18.4 (13.7–23.0)	20.3 (14.4–26.3)	14.6 (7.2–21.7)	0.315
*N*. *gonorrhoeae* (NG)		5.2 (2.6–7.9)	5.6 (2.2–9.1)	4.4 (0.2–8.7)	0.779
*T*. *vaginalis* (TV)		3.0 (1.0–5.0)	1.7 (0–3.6)	5.6 (0.8–10.3)	0.129
CT, NG or TV		23.6 (18.5–28.7)	24.3 (18.0–30.6)	22.2 (13.6–30.8)	0.762
Vaginal flora (Nugent score)	7–10	33.7 (28.0–39.4)	33.3 (26.4–40.3)	34.4 (24.6–44.3)	0.986
	4–6	35.6 (29.8–41.3)	35.6 (28.5–42.6)	35.6 (25.7–45.4)	
	0–3	30.7 (25.2–36.2)	31.1 (24.3–37.9)	30.0 (20.5–39.5)	
Candida		18.0 (13.4–22.6)	19.8 (13.9–25.6)	14.4 (7.2–21.7)	0.316

### EPT uptake among women and their partners

**Fig 1:**
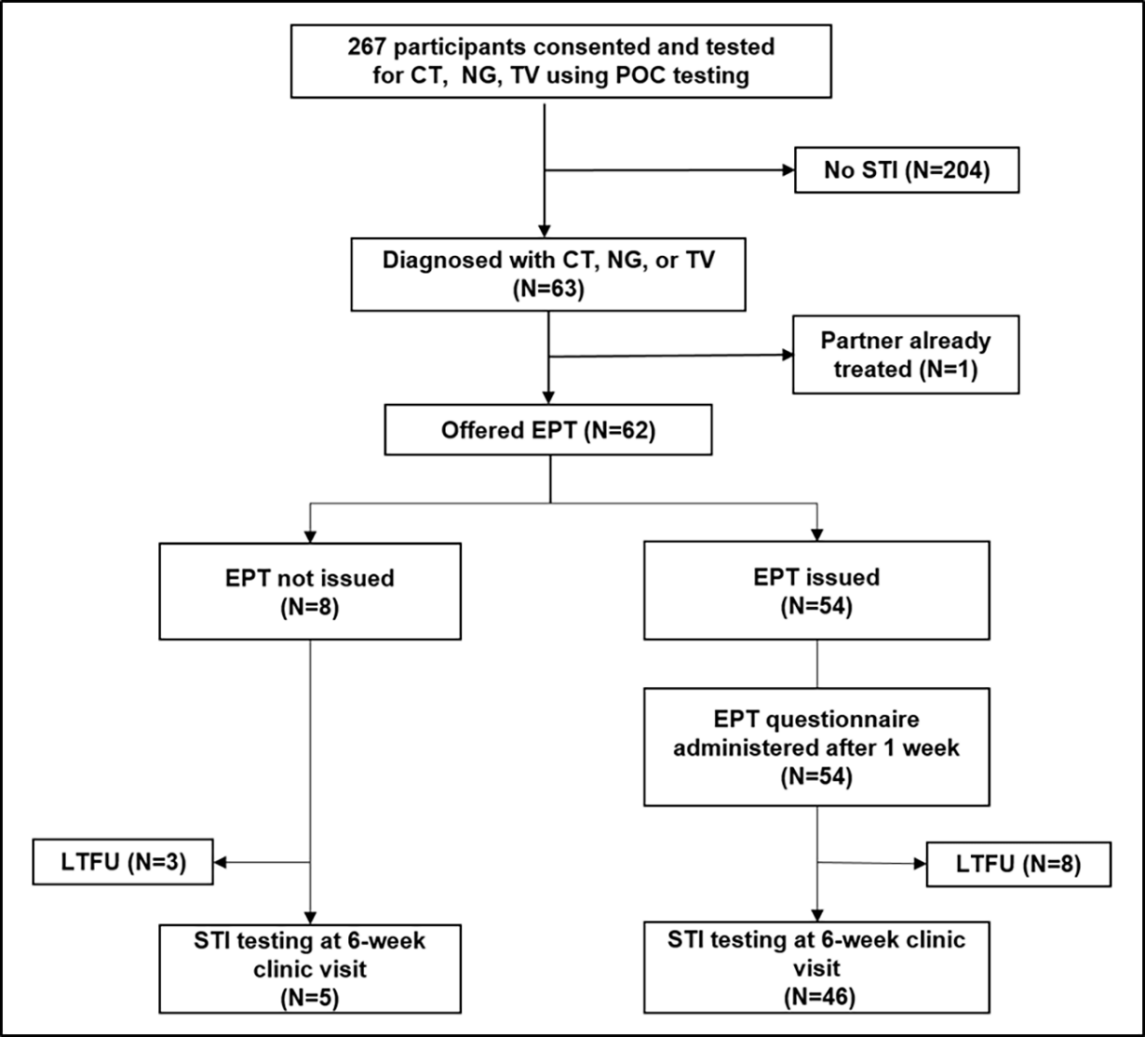
Consort diagram of expedited partner therapy intervention for women in the CAPRISA 083 cohort study. Consenting women were offered participation in the CAPRISA 083 study and underwent point-of-care (POC) testing for sexually transmitted infections (STI). Those diagnosed with Chlamydia (CT), Gonorrhoea (NG) or Trichomoniasis (TV) were offered expedited partner therapy (EPT) to deliver to their partners. Women were contacted by phone one week later, and retested for STIs after six weeks. Some women were lost to follow-up (LTFU).

Of the 63 women with a STI, 98.4% (62/63) were offered and 87.1% (54/62) accepted EPT for their regular partner (Fig 1). The majority (85.7%, 54/63) of women had one partner in the previous two months. Two women chose EPT for one additional casual partner. At telephonic follow-up one week later, 88.9% (48/54) stated that they had successfully delivered EPT, i.e. the partner ingested the medication either observed (77.8%, 42/54) or unobserved (11.1%, 6/54). Five women (9.3%, 5/54) still had to deliver EPT, but managed to deliver it by the 6-week follow-up visit, and one partner refused (1.8%, 1/54). Some women reported that they (16.7%, 9/54) or their partners (6.3%, 3/46) experienced minor medication side effects consistent with the antibiotic profiles, but no allergic reactions or social harms including intimate partner violence were reported. Of the 82.3% (51/62) women completing 6-week follow up, detection rates for any of the three STIs were lower amongst women receiving EPT (2.2%, 1/46), compared to those not receiving EPT (40.0%, 2/5), p = 0.023 (Table 3).

**Table 3:** Comparison of STI detection rates among 51 women with STIs, 6 weeks after an EPT intervention.

Pathogen	Overall (N = 51) % (n/N)	EPT issued (N = 46) % (n/N)	No EPT issued (N = 5) % (n/N)	p-value
*C*. *trachomatis*	3.9 (2/51)	2.2 (1/46)	20.0 (1/5)	0.188
*T*. *vaginalis*	2.0 (1/51)	0	20.0 (1/5)	0.098
*C*. *trachomatis* or *T*. *vaginalis* ^a^	5.9 (3/51)	2.2 (1/46)	40.0 (2/5)	0.023

^a^ No *N*. *gonorrhoeae* cases were detected at 6-week follow-up

### STI detection rates after 6 and 12 weeks

Overall, 37.8% (101/267) women diagnosed with STIs or BV were scheduled for the 6 and 12-week follow-up visits. Retention was 83.2% (84/101) at both 6 and 12 weeks of follow-up with 76.2% (77/101) attending all three clinic visits for retesting (Table 4). In a GEE analysis modelling the effect of time, CT, NG and TV all decreased significantly from baseline with only two cases of CT and one case of NG detected after 12 weeks. Women with BV often required repeat doses of Metronidazole to clear the syndrome. One woman was HIV antibody negative at enrolment, but tested positive at 6 weeks. No other HIV infections were detected during the 12-week follow-up.

**Table 4:** STI detection after POC STI testing, immediate treatment and EPT intervention.

Pathogen (N = 77) ^a^	Baseline N (%)	Week 6 N (%)	Week 12 N (%)	p-value
*C*. *trachomatis* (CT)	35 (45.5)	4 (5.2)	2 (2.6)	<0.001
*N*. *gonorrhoeae* (NG)	10 (13.0)	0 (0)	1 (1.3)	0.041
*T*. *vaginalis* (TV)	5 (6.5)	2 (2.6)	0 (0)	0.013
Any of CT, NG or TV	46 (59.7)	6 (7.8)	3 (3.9)	<0.001
Bacterial vaginosis	40 (52.0)	26 (33.8)	19 (24.7)	<0.001
Nugent Scores mean (IQR)	6.2 (5–7)	5.2 (3–8)	4.8 (3–6)	<0.001
Candidiasis	14 (18.2)	7 (9.1)	12 (15.6)	0.668

^a^ Women with STIs or BV were included if they attended all follow up visits.

### Focus group discussions

A total of 29 women participated in six focus group discussions to assess the acceptability and feasibility of POC STI testing and EPT. Overall, women were relieved to receive a specific STI diagnosis with immediate treatment, which none of them had experienced previously. A specific STI diagnosis meant that women could have a meaningful STI discussion with the nursing staff. Women reported that the STI diagnosis and provision of EPT allowed them to begin communicating with their partners about sexual relationships, and start to renegotiate these, if required. Women reported several outcomes of these discussions including leaving their partners, improved condom use, or continuing their relationships after partner treatment. Overall, women reported that their male sexual partners found EPT helpful for practical reasons, as they did not have to travel to clinics or take time off work to get treated. A few women reported that men got upset about the STI diagnosis or were reluctant to take EPT, but despite this, the majority of men eventually took the EPT. Importantly, women did not report any instances of intimate partner violence as a result of EPT.

## Discussion

This cohort study in young South African women found that an innovative combined model of POC STI testing, immediate treatment, and EPT can be a useful way to reduce the STI burden in LMICs. The study showed that POC STI testing is robust, can be delivered by a laboratory technologist and produces results within 2 hours, a timeframe acceptable to the women. The majority of women accepted EPT and found the EPT pack a useful tool to treat and engage their sexual partners in meaningful discussions about safer sex, condom use, and their relationships. In addition, women who successfully delivered EPT to their sexual partners had statistically lower STI prevalence at follow-up compared to those who did not.

The majority of research on POC STI testing implementation has been conducted in high income countries, including the United States, United Kingdom, and Australia [26,27,28]. The POC tests used in this study were the PCR-based Xpert® CT/NG assay (approximate cost per test USD 14.37) and the OSOM® Rapid Trichomonas Test, an antigen detection assay (approximate cost per test USD 8.00), both of which have been evaluated in multiple settings and achieved high sensitivity and specificity [26,29,30]. Our study showed that these technologies can be used in a LMIC setting, which is encouraging for the development of a workable STI care model, especially in South Africa and other LMICs where the GeneXpert platform has been widely implemented for the rapid diagnosis of Tuberculosis. Additional benefits of this technology include the recent availability of a TV cartridge [31]. In line with other POC molecular diagnostic tests, results currently take several hours to be available, prolonging clinic visit times. Patients in our and other studies did not find this to be problematic, however speedier assays would facilitate patient care and satisfaction [32,33,34].

Our study showed that EPT was acceptable to women, as evidenced by the high uptake, and decreased STI infections at 6 weeks, compared to women who did not take EPT. This comparison was limited by the cohort design of the study and the very small sample of women not taking up EPT. Several other studies have shown that EPT is acceptable to populations and effective in increasing partner treatment and decreasing reinfection rates, but these have primarily been conducted in high income countries [18,35]. However, some studies conducted in LMICs found that EPT was effective in treating partners and acceptable to participants [20,21,36]. In a study from Peru, when participants were offered the choice between concurrent patient partner treatment or EPT, 61.8% chose EPT [36]. Similarly, in a study of female sex workers from Uganda 50.6% accepted EPT for their regular partners. Reported barriers included stigma, fear of being viewed as the primary source of the STI, and loss of contact with sexual partners [37]. Furthermore, similar to our study, research from a different region in South Africa found that EPT was successful in alleviating partners’ fears of visiting or taking time off work to attend a clinic [20].

As confirmed in our study, STIs are common in KwaZulu-Natal, which is also the epicentre of the HIV epidemic, with nearly half of all new HIV infections occurring in 15–24 year-old women [38,39]. The approach of POC testing and immediate treatment led to significant decreases in all STIs at 12 weeks in participants. Treating STIs early and reducing reinfection by treating the sexual partners is therefore particularly important for these young South African women. At a personal level, not only will this improve the sexual and reproductive health of women and their newborns, but it may also reduce their risk of acquiring HIV, by preventing breaches in the mucosal layer or an increase in HIV target cells in the genital tract [40,41]. At a population level, it may have a positive impact on the STI and HIV epidemics.

To the best of our knowledge, this is the first time a model of POC STI testing, immediate treatment, EPT and test of cure has been evaluated in a LMIC setting. Depending on technology innovations, regional epidemiology, and local resources and infrastructure, the individual components of this model could be adapted. For example, given the low detection rates after the intervention, the test of cure could be removed from the model, reducing costs and complexity.

This proof-of-concept study has some limitations including the relatively small sample size, the single urban site setting, and the lack of a cost-effectiveness evaluation. However, this was offset by the fact that the study was conducted by a skilled multi-disciplinary team in a high STI and HIV burden setting, and was able to assess a multi-method approach. While retention rates and EPT uptake were high in the study, this needs to be replicated in a busy clinic setting without dedicated research team support. Finally, some will argue that the feasibility of rolling out EPT at a population level will depend on a more robust surveillance system to detect antibiotic resistance, particularly to NG, in LMICs. Others may argue that these systems should be in place irrespective of the STI care model, and that EPT, by decreasing the overall STI burden, could contribute to keeping antibiotic resistance contained.

Our study suggests that the model of POC STI testing, immediate therapy, EPT and test of cure was acceptable to young South African women and their partners, and resulted in lower STI detection rates on test of cure samples. Larger randomized controlled trials in LMICs should evaluate the feasibility and cost-effectiveness of implementing this strategy at population level, and the effect on STI prevalence and HIV incidence.

## Supporting information

S1 Checklist (PDF)

S1 Fig Expedited partner therapy information leaflet. (DOCX)

S1 Dataset (XLSX)

S1 Study protocol (PDF)

## References

[1] UNAIDS (2016) AIDS by the numbers. Available from: http://www.unaids.org/en/resources/documents/2016/AIDS-by-the-numbers (Accessed 12 April 2018). Geneva, Switzerland: Joint United Nations Programme on HIV/AIDS.

[2] Laga M, Manoka A, Kivuvu M, Malele B, Tuliza M, et al (1993) Non-ulcerative sexually transmitted diseases as risk factors for HIV-1 transmission in women: results from a cohort study. AIDS 7: 95–102. 8442924 10.1097/00002030-199301000-00015

[3] Plummer FA, Simonsen JN, Cameron DW, Ndinya-Achola JO, Kreiss JK, et al (1991) Cofactors in male-female sexual transmission of human immunodeficiency virus type 1. J Infect Dis 163: 233–239. 1988508 10.1093/infdis/163.2.233

[4] Ramjee G, Williams B, Gouws E, Van Dyck E, De Deken B, et al (2005) The impact of incident and prevalent herpes simplex virus-2 infection on the incidence of HIV-1 infection among commercial sex workers in South Africa. J Acquir Immune Defic Syndr 39: 333–339. 15980695 10.1097/01.qai.0000144445.44518.ea

[5] Stamm WE, Handsfield HH, Rompalo AM, Ashley RL, Roberts PL, et al (1988) The association between genital ulcer disease and acquisition of HIV infection in homosexual men. JAMA 260: 1429–1433. 3404600

[6] Wasserheit JN (1992) Epidemiological synergy. Interrelationships between human immunodeficiency virus infection and other sexually transmitted diseases. Sex Transm Dis 19: 61–77. 1595015

[7] Romoren M, Hussein F, Steen TW, Velauthapillai M, Sundby J, et al (2007) Costs and health consequences of chlamydia management strategies among pregnant women in sub-Saharan Africa. Sex Transm Infect 83: 558–566. doi: 10.1136/sti.2007.026930 17932126 10.1136/sti.2007.026930 PMC2598644

[8] Johnston VJ, Mabey DC (2008) Global epidemiology and control of Trichomonas vaginalis. Curr Opin Infect Dis 21: 56–64. doi: 10.1097/QCO.0b013e3282f3d999 18192787 10.1097/QCO.0b013e3282f3d999

[9] Adachi K, Klausner JD, Xu J, Ank B, Bristow CC, et al (2016) Chlamydia trachomatis and Neisseria gonorrhoeae in HIV-infected Pregnant Women and Adverse Infant Outcomes. Pediatr Infect Dis J 35: 894–900. doi: 10.1097/INF.0000000000001199 27164464 10.1097/INF.0000000000001199 PMC4945428

[10] Bosu WK (1999) Syndromic management of sexually transmitted diseases: is it rational or scientific? Trop Med Int Health 4: 114–119. 10206265 10.1046/j.1365-3156.1999.00360.x

[11] Vuylsteke B (2004) Current status of syndromic management of sexually transmitted infections in developing countries. Sex Transm Infect 80: 333–334. doi: 10.1136/sti.2004.009407 15459398 10.1136/sti.2004.009407 PMC1744915

[12] Garrett NJ, McGrath N, Mindel A (2017) Advancing STI care in low/middle-income countries: has STI syndromic management reached its use-by date? Sex Transm Infect 93: 4–5. doi: 10.1136/sextrans-2016-052581 27084840 10.1136/sextrans-2016-052581 PMC5505769

[13] White RG, Moodley P, McGrath N, Hosegood V, Zaba B, et al (2008) Low effectiveness of syndromic treatment services for curable sexually transmitted infections in rural South Africa. Sex Transm Infect 84: 528–534. doi: 10.1136/sti.2008.032011 18708485 10.1136/sti.2008.032011 PMC2584238

[14] Wolday D, GM Z, Mohammed Z, Meles H, Messele T, et al (2004) Risk factors associated with failure of syndromic treatment of sexually transmitted diseases among women seeking primary care in Addis Ababa. Sex Transm Infect 80: 392–394. doi: 10.1136/sti.2003.005660 15459409 10.1136/sti.2003.005660 PMC1744914

[15] Hawkes S, Morison L, Foster S, Gausia K, Chakraborty J, et al (1999) Reproductive-tract infections in women in low-income, low-prevalence situations: assessment of syndromic management in Matlab, Bangladesh. Lancet 354: 1776–1781. 10577639 10.1016/s0140-6736(99)02463-0

[16] White RG, Moodley P, McGrath N, Hosegood V, Zaba B, et al (2008) Low effectiveness of syndromic treatment services for curable sexually transmitted infections in rural South Africa. Sexually transmitted infections 84: 528–534. doi: 10.1136/sti.2008.032011 18708485 10.1136/sti.2008.032011 PMC2584238

[17] Naidoo S, Wand H, Abbai NS, Ramjee G (2014) High prevalence and incidence of sexually transmitted infections among women living in Kwazulu-Natal, South Africa. AIDS Res Ther 11: 31 doi: 10.1186/1742-6405-11-31 25243015 10.1186/1742-6405-11-31 PMC4168991

[18] Golden MR, Whittington WL, Handsfield HH, Hughes JP, Stamm WE, et al (2005) Effect of expedited treatment of sex partners on recurrent or persistent gonorrhea or chlamydial infection. N Engl J Med 352: 676–685. doi: 10.1056/NEJMoa041681 15716561 10.1056/NEJMoa041681

[19] Kissinger P, Hogben M (2011) Expedited partner treatment for sexually transmitted infections: an update. Curr Infect Dis Rep 13: 188–195. doi: 10.1007/s11908-010-0159-3 21365383 10.1007/s11908-010-0159-3

[20] Young T, de Kock A, Jones H, Altini L, Ferguson T, et al (2007) A comparison of two methods of partner notification for sexually transmitted infections in South Africa: patient-delivered partner medication and patient-based partner referral. Int J STD AIDS 18: 338–340. doi: 10.1258/095646207780749781 17524196 10.1258/095646207780749781

[21] Nuwaha F, Kambugu F, Nsubuga PS, Hojer B, Faxelid E (2001) Efficacy of patient-delivered partner medication in the treatment of sexual partners in Uganda. Sex Transm Dis 28: 105–110. 11234783 10.1097/00007435-200102000-00008

[22] Department of Health of South Africa (2010) Gauteng Health Information Management Report. Available from Gauteng Department of Health on request only.

[23] Abdool Karim Q, Abdool Karim SS, Frohlich JA, Grobler AC, Baxter C, et al (2010) Effectiveness and safety of tenofovir gel, an antiretroviral microbicide, for the prevention of HIV infection in women. Science 329: 1168–1174. doi: 10.1126/science.1193748 20643915 10.1126/science.1193748 PMC3001187

[24] Department of Health of South Africa (2015) Sexually Transmitted Infections Management Guidelines 2015 Available from: https://www.idealclinic.org.za/docs/National-Priority-Health-Conditions/Sexually%20Transmitted%20Infections_%20Management%20Guidelines%202015.pdf (Accessed: 12 April 2018). Pretoria: South African Department of Health pp. 1–28.

[25] Centers for Disease Control and Prevention (2015) 2015 Sexually Transmitted Diseases Treatment Guidelines. Available from https://www.cdc.gov/std/tg2015/default.htm (Accessed: 12 April 2018).

[26] Gaydos CA, Van Der Pol B, Jett-Goheen M, Barnes M, Quinn N, et al (2013) Performance of the Cepheid CT/NG Xpert Rapid PCR Test for Detection of Chlamydia trachomatis and Neisseria gonorrhoeae. J Clin Microbiol 51: 1666–1672. doi: 10.1128/JCM.03461-12 23467600 10.1128/JCM.03461-12 PMC3716060

[27] Guy RJ, Natoli L, Ward J, Causer L, Hengel B, et al (2013) A randomised trial of point-of-care tests for chlamydia and gonorrhoea infections in remote Aboriginal communities: Test, Treat ANd GO- the "TTANGO" trial protocol. BMC Infect Dis 13: 485 doi: 10.1186/1471-2334-13-485 24138699 10.1186/1471-2334-13-485 PMC4231474

[28] Atkinson LM, Vijeratnam D, Mani R, Patel R (2016) 'The waiting game': are current chlamydia and gonorrhoea near-patient/point-of-care tests acceptable to service users and will they impact on treatment? Int J STD AIDS 27: 650–655. doi: 10.1177/0956462415591414 26092579 10.1177/0956462415591414

[29] Hegazy MM, El-Tantawy NL, Soliman MM, El-Sadeek ES, El-Nagar HS (2012) Performance of rapid immunochromatographic assay in the diagnosis of Trichomoniasis vaginalis. Diagn Microbiol Infect Dis 74: 49–53. doi: 10.1016/j.diagmicrobio.2012.05.003 22727836 10.1016/j.diagmicrobio.2012.05.003

[30] Nathan B, Appiah J, Saunders P, Heron D, Nichols T, et al (2015) Microscopy outperformed in a comparison of five methods for detecting Trichomonas vaginalis in symptomatic women. Int J STD AIDS 26: 251–256. doi: 10.1177/0956462414534833 24855131 10.1177/0956462414534833

[31] Gaydos CA, Klausner JD, Pai NP, Kelly H, Coltart C, et al (2017) Rapid and point-of-care tests for the diagnosis of Trichomonas vaginalis in women and men. Sex Transm Infect 93: S31–S35. doi: 10.1136/sextrans-2016-053063 28684611 10.1136/sextrans-2016-053063 PMC5723541

[32] Huppert JS, Hesse E, Kim G, Kim M, Agreda P, et al (2010) Adolescent women can perform a point-of-care test for trichomoniasis as accurately as clinicians. Sex Transm Infect 86: 514–519. doi: 10.1136/sti.2009.042168 20595142 10.1136/sti.2009.042168 PMC3221308

[33] Huppert JS, Hesse EA, Bernard MA, Xiao Y, Huang B, et al (2011) Acceptability of self-testing for trichomoniasis increases with experience. Sex Transm Infect 87: 494–500. doi: 10.1136/sextrans-2011-050037 21795289 10.1136/sextrans-2011-050037 PMC3187610

[34] van der Helm JJ, Sabajo LO, Grunberg AW, Morre SA, Speksnijder AG, et al (2012) Point-of-care test for detection of urogenital chlamydia in women shows low sensitivity. A performance evaluation study in two clinics in Suriname. PLoS One 7: e32122 doi: 10.1371/journal.pone.0032122 22393383 10.1371/journal.pone.0032122 PMC3290553

[35] Shiely F, Hayes K, Thomas KK, Kerani RP, Hughes JP, et al (2010) Expedited partner therapy: a robust intervention. Sex Transm Dis 37: 602–607. doi: 10.1097/OLQ.0b013e3181e1a296 20601929 10.1097/OLQ.0b013e3181e1a296

[36] Nguyen M, Cabeza J, Segura E, Garcia PJ, Klausner JD (2016) High Rate of Partner Treatment Among Chlamydia trachomatis-Infected Pregnant Women in Lima, Peru. Sex Transm Dis 43: 296–298. doi: 10.1097/OLQ.0000000000000436 27100765 10.1097/OLQ.0000000000000436 PMC4840465

[37] Mayanja Y, Mukose AD, Nakubulwa S, Omosa-Manyonyi G, Kamali A, et al (2016) Acceptance of Treatment of Sexually Transmitted Infections for Stable Sexual Partners by Female Sex Workers in Kampala, Uganda. PLoS One 11: e0155383 doi: 10.1371/journal.pone.0155383 27171270 10.1371/journal.pone.0155383 PMC4865125

[38] Kharsany AB, Mlotshwa M, Frohlich JA, Yende Zuma N, Samsunder N, et al (2012) HIV prevalence among high school learners—opportunities for schools-based HIV testing programmes and sexual reproductive health services. BMC Public Health 12: 231 doi: 10.1186/1471-2458-12-231 22439635 10.1186/1471-2458-12-231 PMC3359203

[39] Shisana O, Rehle T, Simbayi L, Zuma K, Jooste S, et al (2014) South African National HIV Prevalence, Incidence and Behavioural Survey, 2012 Cape Town: Human Sciences Research Council.

[40] Masson L, Passmore JA, Liebenberg LJ, Werner L, Baxter C, et al (2015) Genital inflammation and the risk of HIV acquisition in women. Clin Infect Dis 61: 260–269. doi: 10.1093/cid/civ298 25900168 10.1093/cid/civ298 PMC4565995

[41] Mlisana K, Naicker N, Werner L, Roberts L, van Loggerenberg F, et al (2012) Symptomatic vaginal discharge is a poor predictor of sexually transmitted infections and genital tract inflammation in high-risk women in South Africa. J Infect Dis 206: 6–14. doi: 10.1093/infdis/jis298 22517910 10.1093/infdis/jis298 PMC3490689

