# Supplementary material for: Beyond syndromic management: Opportunities for diagnosis-based treatment of sexually transmitted infections in low- and middle-income countries
Source: PLoS One. 2018 Apr 24;13(4):e0196209. doi: 10.1371/journal.pone.0196209 (PMC5918163; doi:10.1371/journal.pone.0196209)
Supplement: S1 Fig — (DOCX) [file pone.0196209.s002.docx]

Hello,

As part of a research study at CAPRISA, your partner was recently tested and treated for an infection that could have been spread through sex. You may also have the same infection and would benefit from treatment. In this study we would like to find out whether it is acceptable to provide treatment for you by sending it with your partner to give to you. This is to ensure that you are treated as soon as possible. We do not know your name or your contact details.

This medicine was given to your partner by a doctor to treat you. You may choose between 2 options:

1.
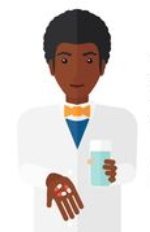
**Take the pills**

If you chose this option, please note:

- It is possible to have an infection without signs and symptoms
- These medicines are antibiotics and are very safe. Side effects are not common. However, if you experience any problems like a rash, trouble breathing or swelling of the lips and tongue, then you may be allergic to the medicines. Please go to your local clinic to get immediate medical assistance.
- After taking these medicines, do not have unprotected sex for at least 7 days.
- Please, do not share these medicines or give them to anyone else.

1. **Alternatively, you can go and see a doctor or nurse at your local clinic for a check-up.**

If you have any questions about the study or are unsure about what to do next, please contact the study nurse on **031 260 1781**.
