# Supplementary material for: Beyond syndromic management: Opportunities for diagnosis-based treatment of sexually transmitted infections in low- and middle-income countries
Source: PLoS One. 2018 Apr 24;13(4):e0196209. doi: 10.1371/journal.pone.0196209 (PMC5918163; doi:10.1371/journal.pone.0196209)
Supplement: S1 Study protocol — (PDF) [file pone.0196209.s004.pdf]

PROTOCOL TITLE:

Proof-of-Concept Study

Changing the STI care model to reduce genital inflammation and HIV risk in South African women

PROTOCOL VERSION 3.0

DATE: 01 MARCH 2016

STUDY SITES:

CAPRISA eThekwini Clinical Research Site, Durban, South Africa
Prince Cyril Zulu Communicable Diseases Clinic, Durban, South Africa

PRINCIPAL INVESTIGATORS:

Dr Nigel Garrett, CAPRISA/UKZN
Prof Anne Rompalo, Johns Hopkins University, US

CO-INVESTIGATORS:

Prof Koleka Mlisana, UKZN
Ms Hope Ngobese, Durban Municipality (DoH)
Dr Lenine Liebenberg, CAPRISA
Mrs Natasha Samsunder, CAPRISA
Dr Kerry Leask, CAPRISA
Prof Adrian Mindel, CAPRISA
Prof Jo-Ann Passmore, CAPRISA/UCT
Dr Andrew Gibbs, HEARD (UKZN)

TABLE OF CONTENTS

1. PROJECT SUMMARY 3
2. BACKGROUND 4
2.1 Significance 4
2.2 Innovation 5
2.3 Preliminary results 6
3. AIM 7
4. OBJECTIVES 7
4.1 Primary Objective 7
4.2 Secondary Objectives 7
4.3 Exploratory Objectives 8
5. STUDY DESIGN 8
5.1 Study population 8
5.2 Inclusion Criteria 8
5.3 Exclusion Criteria 8
5.4 Stage 1: Screening and initial participant assessment 8
5.5 Stage 2: Proof-of-concept study 9
5.5.1 Interventions 9
a) Point-of-Care STI testing 9
b) Immediate index case therapy 9
c) Expedited partner therapy 10
d) Follow-up with Test of Cure 10
5.5.2 Primary study outcome 11
Inflammatory cytokine profiles as indicator of HIV risk in the female genital tract 11
5.5.3 Schedule of evaluation 13
5.5.4 Staff training 13
6. SAFETY 13
6.1 Potential risks and adequate protection against risks 13
6.2 Potential benefits of the proposed research 14
7. PARTICIPANT REIMBURSEMENT 14
8. ANALYSIS 14
9. QUALITY CONTROL AND QUALITY ASSURANCE 15
10. ETHICAL CONSIDERATIONS 15
11. EXPECTED RESULTS AND ALTERNATIVE APPROACHES 16
12. POTENTIAL ROADBLOCKS 16
13. FEASIBILITY 16
14. RELEVANCE 17
15. REFERENCES 17

1. PROJECT SUMMARY

Sexually transmitted infections (STIs) are strongly associated with HIV risk. However, population based studies to manage STIs as a way of reducing HIV risk have had limited success. Recent studies show that elevated genital tract inflammatory cytokines are strongly associated with an increased risk of HIV acquisition, and STIs are one of the commonest causes of elevated genital tract cytokines [1-4]. This interest in the role of cytokines in HIV acquisition has reinvigorated interest in STIs and whether better management strategies can have a role to play in HIV risk reduction.

HIV and STIs are extremely common in the South African province of KwaZulu-Natal, where there are many challenges with STI diagnosis and treatment, including the reliance on syndromic management, an approach based on the recognition of STI syndromes (vaginal discharge, urethral discharge and genital ulceration), followed by treatment targeting the common causes of the syndrome. This syndromic management approach has a low sensitivity and specificity for detecting the most common curable STIs, such as chlamydia, gonorrhoea, trichomoniasis and syphilis. Studies have shown that only 13% of symptomatic STI infections are cured with the STI treatment services currently offered in KwaZulu-Natal. This low cure rate for symptomatic STIs is compounded by the fact that up to 80% of STIs are asymptomatic, which results in a majority of STIs remaining undiagnosed and untreated [5-7]. Other challenges with current STI management include limited partner notification and treatment, resulting in high levels of reinfection. At a population level, the result is that most STIs remain untreated and the burden of STIs within the community remains unchecked.

Our goal is to determine if an innovative, enhanced programme of STI management will result in a higher cure rate and a lower recurrence rate, with a subsequent reduction in genital inflammatory cytokines and hence HIV risk. This proof-of-concept study will identify individuals with STIs using an innovative, point-of-care diagnostic test, an automated, cartridge-based nucleic amplification assay (GeneXpert) for the simultaneous detection of *Neisseria gonorrhoeae* (NG) and *Chlamydia trachomatis* (CT), and *Trichomonas Vaginalis* (TV). This technology has recently been introduced on a large scale across South Africa to detect tuberculosis (TB) and TB drug resistance, thereby accelerating diagnosis, treatment and enhancing public health initiatives to control TB. Genital tract cytokines will be measured using Bio-Plex Pro Human Cytokine kits and a Bio-Plex MagPix Array Reader. Following point-of-care diagnosis, participants will be treated immediately with appropriate therapy under direct supervision, giving the participants the same treatment to take home for their sexual partners (expedited partner therapy) and asking them to return after six weeks and three months for a test of cure and additional cytokine assessments, to determine if these have decreased.

Overall, our innovative enhanced management package for targeted STI care, offers the best opportunity to reduce STIs, by ensuring that the individual is cured and by reducing the risk of reinfection using expedited partner therapy. This will allow us to determine whether genital inflammation can be reduced after effective targeted STI treatment, and ultimately reduce the risk of HIV acquisition in South Africa.

2. BACKGROUND

2.1 Significance

STIs are strongly associated with HIV risk [8-12]. However, population based studies to manage STIs as a way of reducing HIV risk, have had limited success [13-21]. More recent evidence shows that elevated genital tract inflammatory cytokines are strongly associated with an increased risk of HIV acquisition [1, 2]. This interest in the role of cytokines in HIV acquisition has reinvigorated interest in STIs as a potential cause of inflammation, and whether novel management strategies can play a role in HIV risk reduction.

The current evidence linking cytokines to STIs and HIV risk is derived from a handful of studies. A recent study has demonstrated that in patients with CT infection the levels of granulocyte colony stimulating factor (G-CSF), interleukin (IL)-1α, and regulated upon activation normal T cell expressed and presumably secreted (RANTES) were significantly higher in pre-treatment than in post-treatment endocervical secretions, whereas the endocervical levels of IL-10, IL-6, IL-1β, and interferon gamma-induced protein (IP)-10 were also elevated during chlamydial infection, but did not decrease after treatment [22]. Work from our own group has demonstrated that levels of chemokines and other pro-inflammatory cytokines are markedly elevated in women with STIs, whether they are symptomatic or asymptomatic [2]. In addition, several inflammatory cytokines were associated with greater risk of HIV infection. However, a recent study in commercial sex workers in Mombasa, Kenya, failed to confirm this association [23].

The convergent heterosexual epidemics of HIV and STIs are particularly evident in the South African province of KwaZulu-Natal (KZN), which is at the epicentre of the HIV epidemic. In KZN, nearly half of all new HIV infections occur in 15-24 year old women [24, 25], who are up to six times more likely to be infected than their male peers [24, 26, 27]. Although accurate and recent, population-based data are lacking, studies in KZN suggest that STIs are common. A study of 277 women attending either family planning or antenatal clinics in 2002, showed that 23% had TV, 9% CT, 6% NG and 58% bacterial vaginosis (BV) [26].

Throughout South Africa, there are many challenges with STI diagnosis and treatment, including the reliance on syndromic management [28, 29], which has a low sensitivity and specificity for detecting infection [30], particularly for women with vaginal discharge [2]. Syndromic management is an approach based on the recognition of STI associated syndromes (vaginal discharge, urethral discharge and genital ulceration), followed by treatment targeting the common causes of the syndrome [31]. The major problem with this model of care is that 80-85% of individuals with STIs are asymptomatic [2, 32, 33] and as a result, remain undiagnosed and untreated. Other challenges with current STI management include limited partner notification and treatment, and because specific diagnoses are not made, treated individuals do not have a test of cure. At a population level, the result is that most STIs remain untreated and the burden of STIs within the community remains unchecked.

In line with World Health Organization recommendations, syndromic management remains the model of care in resource-limited settings [33]. Reasons for South Africa not moving to a diagnostic treatment model include a lack of skills and expertise, limited diagnostic and treatment facilities, and cost. The failure of STI intervention programmes for HIV prevention may be because, as stated in an editorial commentary in The Journal of Infectious Diseases, *"The failure of this approach…is not because STDs are not critically important. Rather we are simply unable to treat the right infections, with the right drugs at the right time"* [34]. This study aims to address this challenge of managing STIs, thereby reducing inflammatory cytokines and hence HIV risk.

2.2 Innovation

This proof-of-concept study will determine whether a reduction in inflammatory cytokines can be achieved by adopting a strategy of enhanced STI management in a group of women at high risk of HIV infection in Durban, South Africa. The proposed STI management will include a package of innovative interventions, which if successfully implemented, could lead the way for South Africa to transition to a more effective, diagnostic care model for STIs and a possible reduction in HIV incidence (Figure 1).

Figure 1. Expected cytokine reduction and reduction in HIV risk associated with enhanced STI management, compared with standard of care

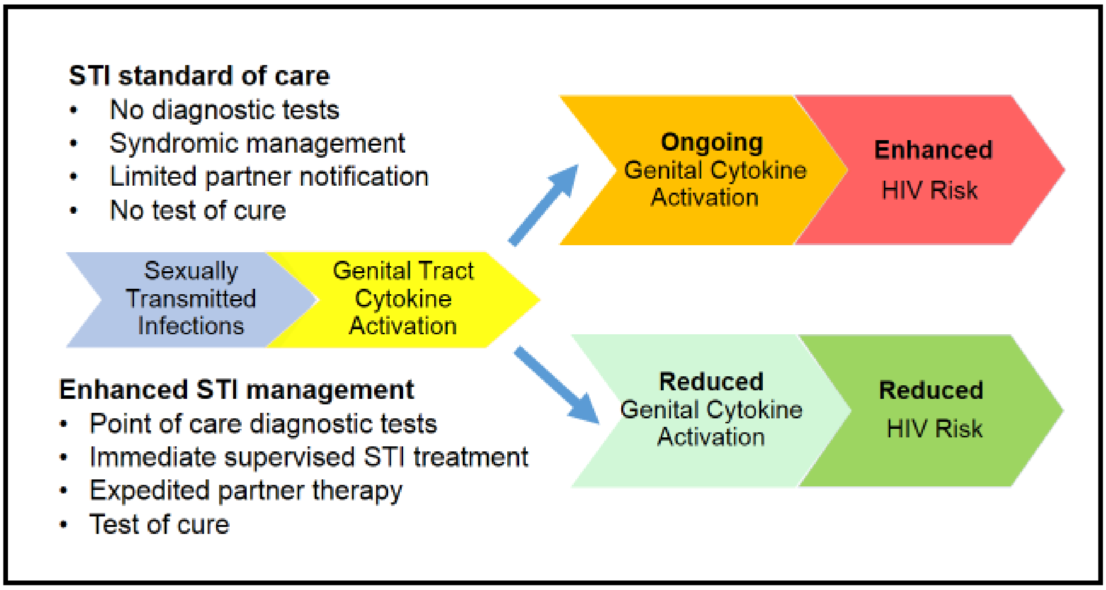

Firstly, we propose to use a new and validated, point-of-care diagnostic platform to determine which infections are present. This will involve an automated, cartridge-based nucleic amplification assay, Xpert® CT/NG and Xpert® TV (Cepheid, Sunnyvale, USA), for the simultaneous detection of CT, NG, and of TV. This technology has recently been introduced on a large scale across South Africa to detect tuberculosis (TB) and TB drug resistance, thereby accelerating diagnosis, treatment and enhancing public health initiatives to control TB. Using the same GeneXpert infrastructure to improve STI care could have a similar impact on public health. As pointed out in a recent review in the New England Journal of Medicine: *"Point-of-care testing can have a transformative effect on health care"* and *"…is expected to expand access to medical services, improve health outcomes, and facilitate the sustainability of disease control programs in low and middle income countries"* [35]. Secondly, we will use evidence-based, single dose regimen to treat the detected STIs and provide directly observed therapy to ensure adherence by the index case. This approach is successfully used in many countries. Thirdly, a key aspect of STI control is to ensure that sexual partners are contacted, tested for STIs and treated. Reliance is usually placed on the patient to inform their sexual partner(s) of an STI diagnosis and refer them to a clinic for treatment. However, this approach has limited success. A variation on this traditional partner notification method is known as expedited partner therapy (EPT), where the index case is provided with a prescription or appropriate medications for their partner(s) [36, 37]. EPT was described as a novel method of partner treatment for STIs as early as 1991, and several randomized trials have demonstrated the superiority of EPT as a partner treatment method when compared with standard partner referral methods [27, 38-42]. The EPT approach has been used in the successful management of both CT and NG, and has been shown to result in fewer reinfections than other methods of partner notification [43]. This method has also been found to be highly acceptable among various study groups, with many patients, in particular adolescents, indicating they prefer EPT to traditional partner notification [44]. Since

the burden of STIs is particularly high among adolescents and young women in KZN, EPT is an important intervention to test within this population. Finally, we propose to introduce a test of cure since we are now able to quickly and accurately assess if a person remains infected using the GeneXpert technology.

Overall, this innovative enhanced management package for STI care, offers the best opportunity to reduce STIs, by ensuring that the individual is cured and by reducing the risk of reinfection using expedited partner therapy. This will allow us to investigate whether genital inflammation can be reduced after effective STI treatment, and ultimately reduce the risk of HIV acquisition in South Africa and other high burden settings.

2.3 Preliminary results

Our study will build on previous STI and cytokine studies conducted at CAPRISA and on the point-of-care test experience from the Johns Hopkins University Point of Care Information Technology Center. CAPRISA has a strong track record of STI research including the epidemiology of STIs, associations with HIV and *herpes simplex virus* type-2 (HSV-2) and syndromic management [2, 10, 26, 45-47]. A recent study, conducted in 160 women who acquired HIV during CAPRISA prevention studies, using nucleic acid amplification assays collected from genital fluid at the acute HIV infection visit, showed that 15.4% had CT, 8.3% NG, and 10.9% TV. More than 70% of these STIs were asymptomatic (Table 1).

Table 1. STIs in 160 women at HIV acquisition

	**% with STI**	**% asymptomatic**
C. trachomatis	15.4	79.2
N. gonorrhoeae	8.3	76.9
T. vaginalis	10.9	88.2
**Bacterial vaginosis**	62.6	78.4

Furthermore, the CAPRISA team has conducted several studies to improve the understanding of the relationship between genital inflammation and HIV infection [48, 49]. We demonstrated that levels of chemokines and other pro-inflammatory cytokines are markedly elevated in women with STIs compared to women without STIs irrespective of symptoms (Figure 2) [2].

Figure 2. Cytokine profiles of women without STIs and women who had asymptomatic or symptomatic infections. Cytokines were grouped into single components using principal component analysis.

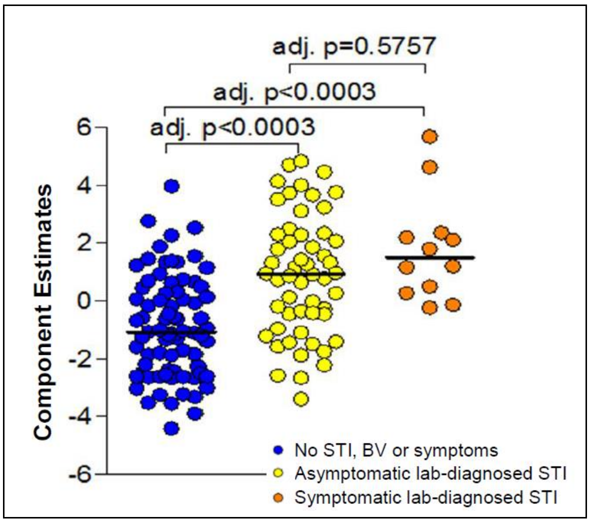

In addition, several inflammatory cytokines, IL-1β, IL-6, IL-8, and soluble CD40 ligand (sCD40L) were associated with greater risk of HIV infection. A study of cervico-vaginal lavage (CVL) samples collected during the CAPRISA 004 microbicide trial [50] showed that 58 women who became HIV-1 infected during the trial had significantly elevated genital tract inflammatory markers prior to HIV infection compared to 58 control women who remained HIV uninfected [1]. This was evidenced by increased concentrations of macrophage inflammatory protein (MIP)-1α, MIP-1β, IL-1α, IL-1β, IL-6, IL-8, IP-10, monocyte chemotactic protein (MCP)-1, GM-CSF and IL-10 and determined by logistic regression and principle component analyses. These associations were independent of study arm, age and other measured HIV risk factors. Genital tract inflammation, defined here as being in the highest quartile for any combination of ≥5 out of 9 inflammatory cytokines (MIP-1α, MIP-1β, IP-10, IL-8, MCP-1, IL-1α, IL-1β, IL-6, and tumour necrosis factor [TNF]-α) was associated with a 3.2-fold increase in the odds of HIV acquisition (Table 2).

Table 2. Association between genital inflammation prior to HIV infection and HIV acquisition in women from the CAPRISA 004 trial

Level of Inflammation	HIV positive	HIV negative
Genital inflammation present	19	6
Genital inflammation absent	39	52
Total N	58	58
Pair-Matched Odds Ratio (95% CI)	3.2 (1.3 – 7.9)	
P-value	0.014	

The CAPRISA Mucosal Immunology Laboratory is currently addressing the contribution of STIs to the degree of cytokine elevation associated with HIV risk by investigating genital inflammation in more than 400 historical CAPRISA 004 specimens (Liebenberg et al., in preparation).

3. AIM

- To determine if a new model of enhanced STI management can reduce genital tract inflammation, and thereby reduce the risk of HIV acquisition.

4. OBJECTIVES

4.1 Primary Objective

- To determine the changes in genital inflammation in women diagnosed with STIs after a diagnostic care intervention

4.2 Secondary Objectives

- To determine the prevalence of laboratory-diagnosed STIs in women presenting to the Prince Cyril Zulu (PCZ) Communicable Diseases Clinic (CDC)
- To validate GeneXpert technology for STI testing against laboratory-based STI assays
- To assess the feasibility and acceptability of GeneXpert implementation for STI management
- To explore the feasibility and acceptability among patients, if possible their partners, and staff of expedited partner therapy interventions, including index case delivered partner therapy

- To determine the direct and indirect costs and potential savings of a diagnostic care model based on GeneXpert STI testing in the PCZ CDC

4.3 Exploratory Objectives

- To characterize the vaginal microbiome of women with and without STIs and with low or high levels of genital inflammation

- To characterize the proteome of women with and without STIs and with low or high levels of genital inflammation

5. STUDY DESIGN

5.1 Study population

Participants will be recruited from the PCZ CDC, a public, open and free access HIV, TB and STI clinic in the Durban Municipality. Women, aged 18 - 40 years, who have a negative point-of-care HIV test on presentation to the STI clinic will be offered participation in the study. Some women, identified during screening, will be excluded from the study.

5.2 Inclusion Criteria

- Age 18 – 40 years
- Female gender
- HIV negative antibody test at enrollment

5.3 Exclusion Criteria

- **HIV positive women**, because HIV could act as an important confounder to genital inflammation, and because this study focuses on HIV prevention
- **Pregnant women**, because the effects of pregnancy on genital tract inflammation have not been fully elucidated.
- **Women who have had antibiotic treatment within the last 7 days**, because baseline genital inflammation measurements could have been affected by treatment.
- **Women who disclose any form of sex work**, because they may be at risk of repeated exposure to STIs and possible ongoing genital tract inflammation during the study period, and may have multiple untraceable sexual contacts.

5.4 Stage 1: Screening and initial participant assessment

Information about the study will be provided by the nurses to Prince Cyril Zulu STI Clinic attendees. Potentially eligible participants identified in the clinic will be provided with additional verbal and written information about the study, and will receive safer sex counselling and condoms. Once consent is obtained, the participants will first have a vaginal swab taken for GeneXpert STI analysis which will take approximately 90 minutes to process. While awaiting the results, a menstrual cup (SoftCup®, EuroFemPro, Netherlands) will be placed to collect genital secretions for future cytokine analysis. While the menstrual cup is in situ and the GeneXpert results are being processed, the participant will be asked to complete a study interviewer administered questionnaire about their sexual behaviour, general and reproductive health. The menstrual cup will then be removed after approximately 1 hour, which will be followed by a genital examination with a Cusco speculum to assess for any signs of STIs, as is currently standard of care in the clinic. In addition, participants will have genital swabs taken for microbiological investigations and to allow assessment of the vaginal proteome and microbiome, as well as a cytobrush to evaluate immune activation markers. . Participants will have a genital swab analysed for CT/ NG and TV making use of the GeneXpert assay and the OSOM® Rapid Trichomonas Test which have already been installed in the clinic laboratory in the same building, and a back-up

machine in the adjacent CAPRISA clinic. While awaiting the GeneXpert result, Gram stain and wet film microscopy will be performed to diagnose BV, Candida and TV. Vaginal candidiasis and BV are important potential confounders when assessing cytokines in the genital tract. Women who test positive for CT, NG, TV or BV will move to the second stage of the study. Women diagnosed with Candida alone will be offered treatment on the day of the visit, but together with women without STIs, will exit the study after the initial assessment. However, collected specimen including Softcup specimen will be stored for future analysis.

5.5 Stage 2: Proof-of-concept study

Women who test positive for CT, NG, TV or BV, will receive enhanced management for the STI(s) or BV and will be followed up in the study for three months. Enhanced management is defined here as the entire novel package of STI interventions which include validated point of care diagnosis, single dose treatment regimen of index case with directly observed therapy, expedited partner therapy and test of cure. This enhanced management method stands in comparison to the current standard of care for STIs in South Africa, which is syndromic management based on diagnosis algorithms followed by treatment to target the symptoms exhibited by the patient.

5.5.1 Interventions

a) Point-of-Care STI testing

The large scale implementation of the Xpert® MTB/RIF, a qualitative real-time PCR test for automated detection of TB in KZN has paved the way to use this technology for the detection of CT, NG and TV. The combined Xpert® CT/NG assay has been evaluated in clinical studies in the USA and Australia, and shows very high sensitivity and specificity for both organisms [51-53]. This assay will be used, and the results will be available within 90 minutes. We will use vaginal swab specimen taken during the clinical examination. The Xpert® TV test has recently been released in the US, and may also be used in this study, or if not available by the time of study start, will be replaced by an alternative rapid Trichomonas test, as well as microscopy. The wet film microscopy method for TV is less sensitive than DNA detection [54-60], but will allow some degree of evaluation of the Xpert® TV assay (or alternative assay). In addition to GeneXpert testing, a vaginal swab will be used for Gram staining for the diagnosis of BV using Nugent’s criteria and for the diagnosis of Candida [61]. The swab will be stained in the clinic and results will be available immediately.

b) Immediate index case therapy

Participants will be treated with single dose therapy, which will be administered under health care worker supervision and, as much as possible, will be in line with South African STI treatment guidelines (Table 3) [62]. Participants diagnosed with NG will be treated with a single 250 mg dose of intramuscular Ceftriaxone as well as Azithromycin 1 gram orally. Resistance to the third generation extended spectrum cephalosporins has been reported from Europe, Asia, North America and South Africa [63-65]. Consequently, participants with NG will have an additional genital swab sample sent for culture and sensitivity taken once the result is available. However, this will not delay treatment. If resistance is noted, the participant will be recalled and treated in line with antibiotic sensitivity testing. Participants with CT will be treated with Azithromycin 1 gram orally in line with the most recent South African STI treatment guidelines [62]. Azithromycin offers single dose treatment and has a similar efficacy to Doxycycline. It is also the first-line treatment in the USA, the United Kingdom and Australia [66, 67]. Participants with TV and BV will be treated with Metronidazole 2 gram orally, in line with South African STI treatment guidelines [62].

Table 3: Proposed Antibiotic therapy for immediate therapy consistent with South African treatment guidelines

Drug	Dosage	Route of Administration	Duration	Diagnosis
Ceftriaxone	250mg	intramuscular	stat	N. Gonorrhoeae
Azithromycin	1g	oral	stat	N. Gonorrhoeae and C. trachomatis
Metronidazole	2g	oral	stat	T. vaginalis and bacterial vaginosis

c) Expedited partner therapy

A key aspect of STI control is to ensure that sexual partners are contacted, tested for STIs and treated appropriately. Many patients are reinfected with an STI if their partner does not receive adequate treatment, which contributes to the high STI prevalence rate. Reliance is usually placed on the patient to inform their sexual partner(s) about the diagnosis of an STI and to refer them for proper treatment and testing. However, this standard approach of partner referral has limited success, so new methods must be developed and implemented to successfully reduce the burden of STIs. One such method is EPT, or patient delivered partner treatment, where the index case is provided with a prescription or appropriate medications for their partner(s) [43]. This approach has been used in the management of CT and NG, and has been shown to result in fewer reinfections than other methods of partner notification [36, 68]. EPT has also been shown to result in a larger proportion of partners being treated as compared to other partner referral methods [38, 39]. High levels of acceptance and adherence to this treatment strategy have been reported in the literature [42, 69]. Before implementation of this intervention the study team will invite women who would meet the eligibility criteria to contribute to focus group discussions on the feasibility and acceptability of this intervention. Depending on findings from these discussions, this intervention could be adapted to include a helpline, or counselor delivered partner notification, if this is found to be more acceptable. All women will be counseled about the importance of ensuring that sexual partner(s) are treated.

Participants diagnosed with NG, CT, and TV during stage 1 or 2 of the study will be provided with a pre-prepared pack for their partner(s), which will include information about safer sex and the STIs, condoms and the appropriate treatment, similar to that of the index case. Contacts of participants diagnosed with NG will be offered Cefixime 400mg orally as a once off dose instead of Ceftriaxone 250mg intramuscularly as well as Azithromycin 1 gram, or have the option to attend in person to the clinic for care. There will be a phone number which will enable the partner to talk directly to a health care provider about treatment and health or relationship concerns they may have. BV is not a STI, although it is associated with sexual activity. Partner notification has not been shown to be important in this condition and will not be undertaken.

d) Follow-up with Test of Cure

All participants will be contacted after one week, to check for any adverse effects from the treatment and to establish if sexual partners were contacted and took the treatment. Participants will be offered the opportunity to talk to a counsellor or nurse if required. Participants will be asked to reattend for a test of cure and a repeat cytokine test after six weeks. The GeneXpert detects the organism’s DNA, which may persist for several weeks after treatment. If the participant is cured and not reinfected, then the test should be negative at six weeks [70]. Participants will all be asked to report to the clinic at three months for cytokine tests and repeat STI and HIV screen. If a participant tests positive for an STI they

will be retreated after checking results on the visits and NG resistance from the previous visit. In the event of a positive HIV result, participants will be assessed and managed according to the South African Adult HIV management guidelines [71], but will be encouraged to complete follow-up in the study.

5.5.2 Primary study outcome

Inflammatory cytokine profiles as indicator of HIV risk in the female genital tract

The concentrations of 48 cytokines including pro-inflammatory cytokines, will be measured in specimen collected from the genital tract using the Softcup device. This device has already been successfully implemented in the CAPRISA 002 Acute Infection study, and has shown to be a reliable method with high acceptability among participants [72]. Cytokines will be measured at baseline, at six weeks and at three months. Based on our previous work and studies published by others on CT, the cytokines of greatest interest are G-CSF, IFN-γ, IL-10, IL-6, IL-8, IL-1α, IL-1β, IP-10, IL-12p70, MCP-1, MIP-1β, MIP-1α, RANTES, TNF-α, TNF-β and vascular endothelial growth factor (VEGF) [1, 2, 22]. Cytokine concentrations will be measured before and after treatment using Bio-Plex Pro Human Cytokine kits and a Bio-Plex MagPix Array Reader (Bio-Rad Laboratories). The sensitivity of these kits range between 0.2 and 45.2pg/ml for each of the 48 cytokines. While the cytokine panel included are not exhaustive, the kits strategically represent the pleiotropic and redundant nature of cytokines by including multiple cytokines involved in cellular recruitment, growth, adaptive responses, regulation, the proinflammatory response or combinations of these (Figure 3), and extends the panels of cytokines previously used to characterise biomarkers of genital inflammation that impact HIV risk [1, 2, 22].

Figure 3. Key functions of cytokines in the Bio-Plex Pro Human Cytokine kits

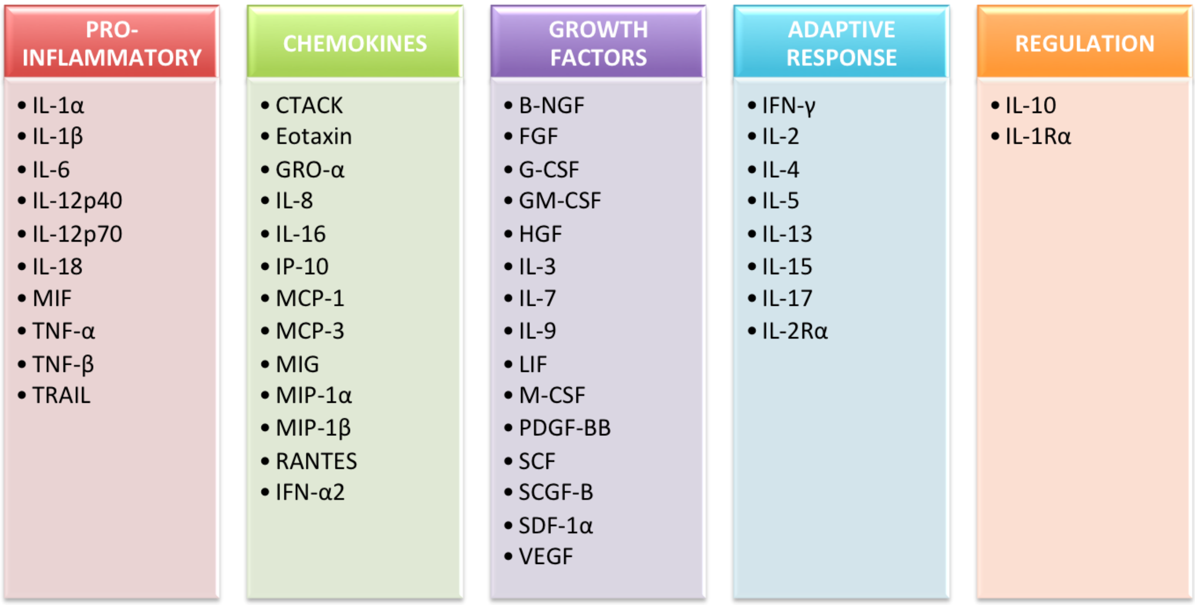

We will use Softcups to collect specimens for the detection of genital tract cytokines as previously described by the study team [72]. A Softcup is a disposable menstrual cup, composed of a sac and a firmer ring from a polymeric material, similar in shape to a diaphragm. The advantages of the Softcup include no sample dilution and no need for a speculum insertion. Softcup specimens will be transported on ice to the CAPRISA laboratory

within 2 hours, where samples will be centrifuged and the pellet and supernatant fractions will be separated and stored at -85˚C for future use.

5.5.3 Secondary study outcomes

Prevalence of laboratory-diagnosed STIs in women presenting to the PCZ CDC
GeneXpert and diagnostic testing of specimen from women attending the PCZ CDC will enable the study team to determine the prevalence of CT, NG and TV in the clinic population. In addition, this information will assist in evaluating the current syndromic management approach in the clinic.

Accuracy of GeneXpert STI testing when compared to laboratory-based PCR assays
The sensitivity and specificity of the GeneXpert STI assay will be compared to a validated laboratory-based PCR assay, the multiplex PCR assay for CT/NG/TV and Mycoplasma genitalium, which was developed in-house by the National Institute for Communicable Diseases in Johannesburg, which is being used in other ongoing CAPRISA studies. New STI assays may also be evaluated as part of this project, if they become available.

Feasibility and acceptability of GeneXpert implementation for STI management
The feasibility of GeneXpert implementation will be assessed throughout study implementation and study conduct. Staff will be asked to provide feedback in the form of a questionnaire, which will be developed prior to the beginning of the study and then altered accordingly once potential advantages and challenges have been identified. Acceptability will be assessed by measuring GeneXpert processing and participant waiting times. Participants and staff will be asked to complete feedback questionnaires to assess the acceptability of diagnostic point-of-care testing in the clinic.

Feasibility and acceptability among patients, their partners and staff of EPT intervention
Cure rate at six weeks and 3 months will be compared between those women who participate in and deliver EPT in the study, and those who do not. In addition, qualitative research will include focus group discussions before and after the EPT intervention. Initial group discussions will focus on potential barriers of patient delivered partner therapy and strategies women think may work to navigate around these barriers. After implementation the focus groups discussions will focus on participants’ experiences of partner notification, whether it increased any experience of risk (e.g. violence or abandonment) and what women would want if they were to do this again. Every effort will be made to invite partners of the index cases, either to attend to the clinic, or to be contacted by phone to discuss their experiences of this intervention. Finally, staff will also have the opportunity to share their experiences.

Direct and indirect costs, and potential savings of a diagnostic care model based on GeneXpert STI testing
While a full cost-effectiveness model is beyond the scope of this pilot study, it will generate financial data, which could form the basis of future cost-effectiveness models. For example, the study will determine staffing requirements to run the GeneXpert assays and assay costs. Any additional hidden costs (e.g. servicing of equipment) and potential cost-savings (e.g. due to improvements to the clinic flow or reductions in repeat attenders) will also be captured. A simple cost-effectiveness model will be presented.

5.5.3 Schedule of evaluation

	Screening and Cohort enrollment (Study entry)	1 week	6 week	3 month (Study exit)
**Locator info**	x	x	x	x
**STI and safe sex counselling**	x	x	x	x
**HIV POC antibody test**	x		x	x
**Pregnancy test**	x		x	x
**Behavioural risk assessment questionnaire**	x		x	x
**Clinical assessment** (x)= only if symptomatic	x		(x)	(x)
**Vaginal swab for GeneXpert CT/NG and TV**	x		x	x
**Swabs for microscopy and storage**	x		x	x
**Softcup and one cytobrush for cytokine assessment**	x		x	x
**Index case therapy**	x		x	x
**Expedited partner therapy**	x		x	x
**Phone follow up**		x		
**Test of Cure**			x	x

5.5.4 Staff training

Prior to the onset of the study, all staff will be trained about the study procedures, processes and documentation. In addition, all staff will be updated regarding the epidemiology, clinical features, diagnosis and treatment of STIs. Particular attention will be paid to the partner notification procedures and the importance of expedited partner therapy.

6. SAFETY

This study will enroll female participants, many of whom are at high risk of acquisition of HIV and STIs. They form a vulnerable group that is heavily affected by HIV and other health issues. Their vulnerability is also compounded by broader social determinants of health and therefore these represent important ethical concerns for participants. However, research in this population over the past two decades has been conducted with relatively little interference from the authorities. This is based largely on a model of community engagement, risk reduction, and reduction of stigma. Epidemiologic data from this province (over 50% STIs and 26% HIV prevalence) indicates that the at-risk population is large, with significant health care needs that include comprehensive HIV prevention and care services, dependable contraception and protection from sexual violence. The programme aspects of this study will address STIs in this community and how enhanced STI management may reduce HIV risk.

6.1 Potential risks and adequate protection against risks

Although the proposed activities pose minimal risks to participants, confidentiality will continue to be a priority and will be protected. This includes, storing all data forms in secure lockers after entry into an encrypted database with controlled access, stored data will be password protected, and program staff will be trained on research ethics. In addition, all participant interviews, focus group discussion, and surveys will take place in privacy and in appropriate locations. A unique identification system will be used to protect participant information, and at no time will the participant identifiers be shared with non-program staff. Participants who become pregnant will be referred to appropriate ante-natal care

programmes. Most importantly, any individual who wishes not to participate in research will still be able to access all of the program services without any issue.

All data generated by this project will be shared via peer-reviewed publications in a timely fashion. To protect individual patients, no identifying information will be used and reports and publication will include only summary statistics.

6.2 Potential benefits of the proposed research

The participants will benefit from the study in several ways. Firstly, potential participants will be screened for STIs using point-of-care tests (the standard of care is syndromic management) and will be provided with appropriate antimicrobial treatment for themselves and their sexual contacts free of charge. Treatment may reduce the risk of pelvic inflammatory disease, chronic pelvic pain, infertility and ectopic pregnancies. In addition, all potential participants will be tested for HIV and those who are positive will be immediately referred for appropriate clinical care. Those who are HIV negative will benefit from receipt of HIV prevention services. However, the broader relevance of these data will hopefully inform policy and improve biomedical HIV prevention efforts, which represents a potential indirect benefit to society at large, even though this is a complex long-term research goal. Even if the hypotheses of the proposed research are found to be incorrect, the knowledge gained from negative data will still be important in moving the HIV field forward.

7. PARTICIPANT REIMBURSEMENT

Study participants will be reimbursed as per CAPRISA Policy on Study Participant Compensation guidance considering time of visits, inconvenience of study procedures, and transport expenses. It is anticipated that the reimbursement per screening or follow-up visit will be approximately ZAR 150.

8. ANALYSIS

Demographic and baseline behavioral characteristics will be described. Cytokine concentrations will be log transformed to ensure normality. Paired t-tests will be performed comparing cytokine concentrations before and after STI treatment, i.e. baseline compared to 6 weeks. Additional analysis will be performed comparing the 12 week follow-up time point to the baseline and 6 week measurements. Paired t-tests will be performed for each STI and BV and any STI, comparing baseline cytokine measurements with that measured at 6 and 12 weeks, and general linear models will be used to adjust for other STIs the participants may have. The false discovery rate method will be used to correct for multiple comparisons. In addition, longitudinal multivariate modelling approaches, which accommodate the correlation between cytokine concentrations, will be used to assess the changes in cytokine concentrations over time [73]. Data will be analyzed using SAS version 9.3 (SAS Institute Inc., Cary) and R.

The accuracy of GeneXpert STI testing will be assessed using sensitivity and specificity measures compared to the standard multiplex PCR assay for CT/NG/TV and Mycoplasma genitalium that is being used in other CAPRISA studies.

The feasibility and acceptability of GeneXpert will be determined through the administration of questionnaires to staff and participants. The quantitative variables measured in the questionnaire will be summarized and mean outcomes will be compared using t-tests, while medians will be compared using the Wilcoxon rank sum test. Categorical variables will be compared using Fisher’s exact test.

When determining cure rates, the date of cure will be estimated as the midpoint between the last positive STI test date and the first negative STI test date. The focus group discussions

will be analysed for emerging themes in order to determine the feasibility and acceptability of expedited partner therapy interventions.

Sample size calculations

The sample size is based on previous work on cytokine production associated with CT, as this is one of the commonest STIs and the cytokine patterns associated with this STI have been well quantified. The sample size was calculated to determine the minimum sample size required for a 50% reduction in mean cytokine levels after the intervention. We assume a power of 80%, alpha = 0.05 and a conservative estimate of the standard deviation as equal to the mean cytokine level in women prior to the intervention. Two scenarios were chosen for the correlation between the paired observations: moderate (ρ=0.6) and strong (ρ=0.8). Given that these would be in CT positive participants only, this will need to be adjusted for the proportion of women we expect to test positive for STIs to determine the number of women who will be screened. A loss to follow up rate of 10% was included (Table 4).

**Table 4. Sample Size Calculations**			**Number to be screened, given the proportion of STIs in the population**		
**Correlation between paired data**	**Number of pairs**	**Number of pairs** (including loss to follow up)	7.5%	10.0%	12.5%
**Moderate (ρ=0.60)**	28	32	427	320	256
**Strong (ρ=0.80)**	15	17	227	170	136

Based on these calculations we will screen a maximum of 427 participants. This will provide us with an estimated 32 CT positive participants. As our recruitment will be based in a STI clinic, frequented by clients at high risk for STIs, we expect that the prevalence of CT will be well over 7.5%. In order to minimize screening we will stop recruitment when we have reached 32. We expect the prevalence of NG to be ≥7.5%, TV >20% and BV > 50%, so we will recruit all participants with NG, but only a sub-sample of participants with TV and BV. A prevalence study is about to be conducted in the PCZ CDC, which will provide us with accurate prevalence data to refine our sample size calculations before commencement. The clinic sees up to 40,000 patients with new syndromic STD per year, and we therefore do not anticipate challenges with reaching these participant numbers.

9. QUALITY CONTROL AND QUALITY ASSURANCE

CAPRISA has a long track record of conducting quality research. This study will be monitored by the CAPRISA Quality Assurance Team, which will include 6-monthly reports on study conduct findings. Particular attention will be placed on consent forms, adherence to the protocol, completion of source documents and CRFs, and appropriate and timely communication with the ethics committee, especially in the case of any protocol deviation. In addition, the study team will have weekly meetings to monitor study progress. CAPRISA routinely measure QC and retention rates on all studies.

10. ETHICAL CONSIDERATIONS

Collection and analyses of socio-demographic information, reproductive history, sexual risk taking behaviors, and biological samples taken for clinical purposes will be covered by ethics approval. No investigational products will be utilized in this study and hence there is no requirement for regulatory approval from the South African Medicines Control Council. IRB

approval will be sought from the University of KwaZulu-Natal (UKZN) Biomedical Research Ethics Committee (BREC) (FWA00000678). Informed consent will be obtained from potential participants to participate in the study and to store genital and blood samples to test for STIs and genital tract cytokines collected at screening and follow-up visits. These biological specimens will be handled by trained staff and labeled with a unique identifier for each participant. The results will be transmitted to the clinics in an electronic format but only accessed by authorized and trained clinic staff. Only key members of the data team will have access to the electronic data, which will be password protected. Once enrolled into the study participants will be able to withdraw at any point. This will not have a detrimental impact on their care by the study team at the PCZ CDC and CAPRISA clinics.

11. EXPECTED RESULTS AND ALTERNATIVE APPROACHES

We expect to find an overall decline in cytokine levels after treatment in the participants. In addition, we expect to recruit sufficient participants to study the effect of enhanced STI treatment with each of the STIs. Even if we are unable to demonstrate a reduction in cytokine levels, the data generated for the study will provide valuable information about cytokines and STIs and this will be useful in generating hypotheses for new studies. In addition, we will have information that will allow us to evaluate the use of GeneXpert for STI detection and expedited partner therapy in a high prevalence, low resource setting.

12. POTENTIAL ROADBLOCKS

A potential challenge could be the implementation of **expedited partner therapy**. Participant concerns may include compromising confidentiality, difficulties broaching the subject with sexual partners, and concerns that this could provoke partner violence or the partner may leave. In order to address these concerns and develop culturally appropriate processes, we will conduct a series of focus groups with potential recruits and staff members coordinated by a skilled facilitator. Follow-up focus groups with participants will assess whether our approach has increased any potential risks for these women.

Other potential challenges include: 1) **Loss to follow-up**: In order to improve participant compliance and follow up, all participants will be counselled by the research nurse and will have mobile phone access to the clinic staff. In addition, we have accounted for this in our sample size calculations. 2) **Anxiety about the use of the Softcup**: This will be minimised through counselling and advice about Softcup insertion. We have evaluated the Softcup for specimen collection and it was preferred by 100% of participants compared to the speculum examination. 3) **Allergies to therapy**: We will be using widely used medications that have a low allergenic potential and are recommended by the most recent South African STI Management Guidelines. 4) **Pregnancy during follow up**: Participants will be counselled about safer sex and the use of contraception and, if found to be pregnant, will be referred to antenatal care.

13. FEASIBILITY

Participants will be recruited from the STI clinic situated at the PCZ Communicable Disease Centre, which is located in central Durban in the transport hub for public commuters by rail, bus or minibus taxis. Annually, approximately 40 000 cases of STIs are diagnosed syndromically and treated at this clinic. The clinic has the added advantage of housing a laboratory which facilitates rapid sample processing, and on site microscopy. The CAPRISA eThekwini research facility is adjacent to the clinic. CAPRISA has a vast experience in recruiting and retaining participants for clinical studies with a highly trained staff of doctors, nurses, counsellors and pharmacists. Investigation of genital cytokines by Luminex multiplex assays have long been conducted as part of a collaboration between CAPRISA and the

University of Cape Town (UCT) [1, 2, 48, 49]. The CAPRISA Mucosal Immunology Laboratory now conducts genital cytokine assessments in-house, as it is equipped with both a Bio-Plex 200 and a Mag-Pix array reader, and several post-doctoral researchers and technicians trained by UCT and international collaborators to conduct reliable and reproducible assessments of cytokine concentrations. The group at John Hopkins has over 30 years of experience evaluating and implementing new STI diagnostic methods and are currently involved in a project to determine ideal characteristics of STI point-of-care tests. Finally, Dr Andrew Gibbs from Health Economics and HIV and AIDS Research Division (HEARD) at the University of KwaZulu-Natal is one of the leading international experts in qualitative research on gender-based violence in South Africa, and will be supervising the focus group work on EPT.

14. RELEVANCE

This proof of concept study will determine whether a reduction in inflammatory cytokines can be achieved by adopting a novel strategy of enhanced STI management in a group of women at high risk of HIV. This is particularly relevant in KZN which is at the epicentre of the HIV epidemic. The proposed STI management will include a package of highly innovative interventions, which, if successfully implemented, could lead the way for South Africa to transition to a more effective, diagnostic care model for STIs leading to a reduction in HIV incidence.

15. REFERENCES

1. Masson L, Passmore JA, Liebenberg LJ, Werner L, Baxter C, Arnold KB*, et al.* Genital inflammation and the risk of HIV acquisition in women. *Clin Infect Dis* 2015.
2. Mlisana K, Naicker N, Werner L, Roberts L, van Loggerenberg F, Baxter C*, et al.* Symptomatic vaginal discharge is a poor predictor of sexually transmitted infections and genital tract inflammation in high-risk women in South Africa. *J Infect Dis* 2012,**206**:6-14.
3. Crowley-Nowick PA, Ellenberg JH, Vermund SH, Douglas SD, Holland CA, Moscicki AB. Cytokine profile in genital tract secretions from female adolescents: impact of human immunodeficiency virus, human papillomavirus, and other sexually transmitted pathogens. *J Infect Dis* 2000,**181**:939-945.
4. Hel Z, McGhee JR, Mestecky J. HIV infection: first battle decides the war. *Trends Immunol* 2006,**27**:274-281.
5. Korenromp EL, Sudaryo MK, de Vlas SJ, Gray RH, Sewankambo NK, Serwadda D*, et al.* What proportion of episodes of gonorrhoea and chlamydia becomes symptomatic? *Int J STD AIDS* 2002,**13**:91-101.
6. White RG, Moodley P, McGrath N, Hosegood V, Zaba B, Herbst K*, et al.* Low effectiveness of syndromic treatment services for curable sexually transmitted infections in rural South Africa. *Sex Transm Infect* 2008,**84**:528-534.
7. Wilkinson D, Abdool Karim SS, Harrison A, Lurie M, Colvin M, Connolly C*, et al.* Unrecognized sexually transmitted infections in rural South African women: a hidden epidemic. *Bull World Health Organ* 1999,**77**:22-28.
8. Laga M, Manoka A, Kivuvu M, Malele B, Tuliza M, Nzila N*, et al.* Non-ulcerative sexually transmitted diseases as risk factors for HIV-1 transmission in women: results from a cohort study. *AIDS* 1993,**7**:95-102.
9. Plummer FA, Simonsen JN, Cameron DW, Ndinya-Achola JO, Kreiss JK, Gakinya MN*, et al.* Cofactors in male-female sexual transmission of human immunodeficiency virus type 1. *J Infect Dis* 1991,**163**:233-239.
10. Ramjee G, Williams B, Gouws E, Van Dyck E, De Deken B, Karim SA. The impact of incident and prevalent herpes simplex virus-2 infection on the incidence of HIV-1

infection among commercial sex workers in South Africa. *J Acquir Immune Defic Syndr* 2005,**39**:333-339.
11. Stamm WE, Handsfield HH, Rompalo AM, Ashley RL, Roberts PL, Corey L. The association between genital ulcer disease and acquisition of HIV infection in homosexual men. *JAMA* 1988,**260**:1429-1433.
12. Wasserheit JN. Epidemiological synergy. Interrelationships between human immunodeficiency virus infection and other sexually transmitted diseases. *Sex Transm Dis* 1992,**19**:61-77.
13. Celum C, Wald A, Hughes J, Sanchez J, Reid S, Delany-Moretlwe S*, et al.* Effect of aciclovir on HIV-1 acquisition in herpes simplex virus 2 seropositive women and men who have sex with men: a randomised, double-blind, placebo-controlled trial. *Lancet* 2008,**371**:2109-2119.
14. Celum C, Wald A, Lingappa JR, Magaret AS, Wang RS, Mugo N*, et al.* Acyclovir and transmission of HIV-1 from persons infected with HIV-1 and HSV-2. *N Engl J Med* 2010,**362**:427-439.
15. Ghys PD, Diallo MO, Ettiegne-Traore V, Satten GA, Anoma CK, Maurice C*, et al.* Effect of interventions to control sexually transmitted disease on the incidence of HIV infection in female sex workers. *AIDS* 2001,**15**:1421-1431.
16. Gregson S, Adamson S, Papaya S, Mundondo J, Nyamukapa CA, Mason PR*, et al.* Impact and process evaluation of integrated community and clinic-based HIV-1 control: a cluster-randomised trial in eastern Zimbabwe. *PLoS Med* 2007,**4**:e102.
17. Grosskurth H, Mosha F, Todd J, Mwijarubi E, Klokke A, Senkoro K*, et al.* Impact of improved treatment of sexually transmitted diseases on HIV infection in rural Tanzania: randomised controlled trial. *Lancet* 1995,**346**:530-536.
18. Kamali A, Quigley M, Nakiyingi J, Kinsman J, Kengeya-Kayondo J, Gopal R*, et al.* Syndromic management of sexually-transmitted infections and behaviour change interventions on transmission of HIV-1 in rural Uganda: a community randomised trial. *Lancet* 2003,**361**:645-652.
19. Kaul R, Kimani J, Nagelkerke NJ, Fonck K, Ngugi EN, Keli F*, et al.* Monthly antibiotic chemoprophylaxis and incidence of sexually transmitted infections and HIV-1 infection in Kenyan sex workers: a randomized controlled trial. *JAMA* 2004,**291**:2555-2562.
20. Watson-Jones D, Weiss HA, Rusizoka M, Changalucha J, Baisley K, Mugeye K*, et al.* Effect of herpes simplex suppression on incidence of HIV among women in Tanzania. *N Engl J Med* 2008,**358**:1560-1571.
21. Wawer MJ, Sewankambo NK, Serwadda D, Quinn TC, Paxton LA, Kiwanuka N*, et al.* Control of sexually transmitted diseases for AIDS prevention in Uganda: a randomised community trial. Rakai Project Study Group. *Lancet* 1999,**353**:525-535.
22. Sperling R, Kraus TA, Ding J, Veretennikova A, Lorde-Rollins E, Singh T*, et al.* Differential profiles of immune mediators and in vitro HIV infectivity between endocervical and vaginal secretions from women with Chlamydia trachomatis infection: a pilot study. *J Reprod Immunol* 2013,**99**:80-87.
23. Lajoie J, Kimani M, Plummer FA, Nyamiobo F, Kaul R, Kimani J*, et al.* Association of sex work with reduced activation of the mucosal immune system. *J Infect Dis* 2014,**210**:319-329.
24. Kharsany AB, Mlotshwa M, Frohlich JA, Yende Zuma N, Samsunder N, Abdool Karim SS*, et al.* HIV prevalence among high school learners - opportunities for schools-based HIV testing programmes and sexual reproductive health services. *BMC Public Health* 2012,**12**:231.
25. Shisana O, Rehle, T, Simbayi LC, Zuma, K, Jooste, S, Zungu N, Labadarios, D,, Onoya Dea. South African National HIV Prevalence, Incidence and Behavioural Survey, 2012. In. Cape Town: Human Sciences Research Council; 2014.
26. Frohlich JA, Abdool Karim Q, Mashego MM, Sturm AW, Abdool Karim SS. Opportunities for treating sexually transmitted infections and reducing HIV risk in rural South Africa. *J Adv Nurs* 2007,**60**:377-383.

27. Rehle T, Shisana O, Pillay V, Zuma K, Puren A, Parker W. National HIV incidence measures--new insights into the South African epidemic. *S Afr Med J* 2007,**97**:194-199.
28. Lewis DA ME. Revision of the national guideline for first-line comprehensive management and control of sexually transmitted infections: what's new and why? *South Afr J Epidemiol Infect* 2009,**24**:6-9.
29. Pettifor A, Walsh J, Wilkins V, Raghunathan P. How effective is syndromic management of STDs?: A review of current studies. *Sex Transm Dis* 2000,**27**:371-385.
30. Mayaud P, Mabey D. Approaches to the control of sexually transmitted infections in developing countries: old problems and modern challenges. *Sex Transm Infect* 2004,**80**:174-182.
31. Veldhuijzen NJ, van Steijn M, Nyinawabega J, Kestelyn E, Uwineza M, Vyankandondera J*, et al.* Prevalence of sexually transmitted infections, genital symptoms and health-care seeking behaviour among HIV-negative female sex workers in Kigali, Rwanda. *Int J STD AIDS* 2013,**24**:139-143.
32. Allsworth JE, Ratner JA, Peipert JF. Trichomoniasis and other sexually transmitted infections: results from the 2001-2004 National Health and Nutrition Examination Surveys. *Sex Transm Dis* 2009,**36**:738-744.
33. Steen R, Wi TE, Kamali A, Ndowa F. Control of sexually transmitted infections and prevention of HIV transmission: mending a fractured paradigm. *Bull World Health Organ* 2009,**87**:858-865.
34. Cohen MS. Classical sexually transmitted diseases drive the spread of HIV-1: back to the future. *J Infect Dis* 2012,**206**:1-2.
35. Jani IV, Peter TF. How point-of-care testing could drive innovation in global health. *N Engl J Med* 2013,**368**:2319-2324.
36. Estcourt C, Sutcliffe L, Cassell J, Mercer CH, Copas A, James L*, et al.* Can we improve partner notification rates through expedited partner therapy in the UK? Findings from an exploratory trial of Accelerated Partner Therapy (APT). *Sex Transm Infect* 2012,**88**:21-26.
37. Estcourt CS, Sutcliffe LJ, Copas A, Mercer CH, Roberts TE, Jackson LJ*, et al.* Developing and testing accelerated partner therapy for partner notification for people with genital Chlamydia trachomatis diagnosed in primary care: a pilot randomised controlled trial. *Sex Transm Infect* 2015.
38. Golden MR, Whittington WL, Handsfield HH, Hughes JP, Stamm WE, Hogben M*, et al.* Effect of expedited treatment of sex partners on recurrent or persistent gonorrhea or chlamydial infection. *N Engl J Med* 2005,**352**:676-685.
39. Kissinger P, Hogben M. Expedited partner treatment for sexually transmitted infections: an update. *Curr Infect Dis Rep* 2011,**13**:188-195.
40. Nuwaha F, Kambugu F, Nsubuga PS, Hojer B, Faxelid E. Efficacy of patient-delivered partner medication in the treatment of sexual partners in Uganda. *Sex Transm Dis* 2001,**28**:105-110.
41. Ramstedt K, Forssman L, Johannisson G. Contact tracing in the control of genital Chlamydia trachomatis infection. *Int J STD AIDS* 1991,**2**:116-118.
42. Schillinger JA, Kissinger P, Calvet H, Whittington WL, Ransom RL, Sternberg MR*, et al.* Patient-delivered partner treatment with azithromycin to prevent repeated Chlamydia trachomatis infection among women: a randomized, controlled trial. *Sex Transm Dis* 2003,**30**:49-56.
43. Trelle S, Shang A, Nartey L, Cassell JA, Low N. Improved effectiveness of partner notification for patients with sexually transmitted infections: systematic review. *BMJ* 2007,**334**:354.
44. Radovic A, Burstein GR, Marshal MP, Murray PJ, Miller E, Sucato GS. Adolescents' attitudes toward expedited partner therapy for sexually transmitted infections. *Sex Transm Dis* 2013,**40**:894-897.

45. Abdool Q, Karim. Barriers to preventing human immunodeficiency virus in women: experiences from KwaZulu-Natal, South Africa. *J Am Med Womens Assoc* 2001,**56**:193-196.
46. Karim QA, Kharsany AB, Frohlich JA, Werner L, Mashego M, Mlotshwa M*, et al.* Stabilizing HIV prevalence masks high HIV incidence rates amongst rural and urban women in KwaZulu-Natal, South Africa. *Int J Epidemiol* 2011,**40**:922-930.
47. Kharsany AB, Mashego M, Mdlotshwa M, Frohlich J, Karim QA. Direct questioning of genital symptoms: increasing opportunities for identifying and treating sexually transmitted infections in primary health-care settings. *Afr J Reprod Health* 2006,**10**:105-112.
48. Roberts L, Passmore JA, Mlisana K, Williamson C, Little F, Bebell LM*, et al.* Genital tract inflammation during early HIV-1 infection predicts higher plasma viral load set point in women. *J Infect Dis* 2012,**205**:194-203.
49. Roberts L, Passmore JA, Williamson C, Little F, Bebell LM, Mlisana K*, et al.* Plasma cytokine levels during acute HIV-1 infection predict HIV disease progression. *AIDS* 2010,**24**:819-831.
50. Abdool Karim Q, Abdool Karim SS, Frohlich JA, Grobler AC, Baxter C, Mansoor LE*, et al.* Effectiveness and safety of tenofovir gel, an antiretroviral microbicide, for the prevention of HIV infection in women. *Science* 2010,**329**:1168-1174.
51. Dize L, West SK, Mkocha H, Quinn TC, Gaydos CA. Evaluation of pooled ocular and vaginal swabs by the Cepheid GeneXpert CT/NG assay for the detection of Chlamydia trachomatis and Neisseria gonorrhoeae compared to the GenProbe Aptima Combo 2 Assay. *Diagn Microbiol Infect Dis* 2015,**81**:102-104.
52. Gaydos CA. Review of use of a new rapid real-time PCR, the Cepheid GeneXpert(R) (Xpert) CT/NG assay, for Chlamydia trachomatis and Neisseria gonorrhoeae: results for patients while in a clinical setting. *Expert Rev Mol Diagn* 2014,**14**:135-137.
53. Gaydos CA, Van Der Pol B, Jett-Goheen M, Barnes M, Quinn N, Clark C*, et al.* Performance of the Cepheid CT/NG Xpert Rapid PCR Test for Detection of Chlamydia trachomatis and Neisseria gonorrhoeae. *J Clin Microbiol* 2013,**51**:1666-1672.
54. Adu-Sarkodie Y, Opoku BK, Danso KA, Weiss HA, Mabey D. Comparison of latex agglutination, wet preparation, and culture for the detection of Trichomonas vaginalis. *Sex Transm Infect* 2004,**80**:201-203.
55. Cohen CE, Gilmour C, Mandalia S, McLean KA. Microscopy and culture for Trichomonas vaginalis: are both required? *Int J STD AIDS* 2006,**17**:418-420.
56. Ohlemeyer CL, Hornberger LL, Lynch DA, Swierkosz EM. Diagnosis of Trichomonas vaginalis in adolescent females: InPouch TV culture versus wet-mount microscopy. *J Adolesc Health* 1998,**22**:205-208.
57. Radonjic IV, Dzamic AM, Mitrovic SM, Arsic Arsenijevic VS, Popadic DM, Kranjcic Zec IF. Diagnosis of Trichomonas vaginalis infection: The sensitivities and specificities of microscopy, culture and PCR assay. *Eur J Obstet Gynecol Reprod Biol* 2006,**126**:116-120.
58. Schwebke JR, Morgan SC, Pinson GB. Validity of self-obtained vaginal specimens for diagnosis of trichomoniasis. *J Clin Microbiol* 1997,**35**:1618-1619.
59. Thomason JL, Gelbart SM, Sobun JF, Schulien MB, Hamilton PR. Comparison of four methods to detect Trichomonas vaginalis. *J Clin Microbiol* 1988,**26**:1869-1870.
60. van Der Schee C, van Belkum A, Zwijgers L, van Der Brugge E, O'Neill E L, Luijendijk A*, et al.* Improved diagnosis of Trichomonas vaginalis infection by PCR using vaginal swabs and urine specimens compared to diagnosis by wet mount microscopy, culture, and fluorescent staining. *J Clin Microbiol* 1999,**37**:4127-4130.
61. Nugent RP, Krohn MA, Hillier SL. Reliability of diagnosing bacterial vaginosis is improved by a standardized method of gram stain interpretation. *J Clin Microbiol* 1991,**29**:297-301.
62. Africa DoHoS. Sexually Transmitted Infections Management Guidelines 2015. In; 2015. pp. 1-28.

63. Lewis DA. Global resistance of Neisseria gonorrhoeae: when theory becomes reality. *Curr Opin Infect Dis* 2014,**27**:62-67.
64. Lewis DA, Sriruttan C, Muller EE, Golparian D, Gumede L, Fick D*, et al.* Phenotypic and genetic characterization of the first two cases of extended-spectrum-cephalosporin-resistant Neisseria gonorrhoeae infection in South Africa and association with cefixime treatment failure. *J Antimicrob Chemother* 2013,**68**:1267-1270.
65. Unemo M, Golparian D, Nicholas R, Ohnishi M, Gallay A, Sednaoui P. High-level cefixime- and ceftriaxone-resistant Neisseria gonorrhoeae in France: novel penA mosaic allele in a successful international clone causes treatment failure. *Antimicrob Agents Chemother* 2012,**56**:1273-1280.
66. Lazaro N. Sexually Transmitted Infections in Primary Care 2013. In; 2014.
67. Prevention CfDCa. Sexually transmitted diseases treatmetn guidelines. *MMWR Recommendations & Reports* 2010:1-110.
68. Shiely F, Hayes K, Thomas KK, Kerani RP, Hughes JP, Whittington WL*, et al.* Expedited partner therapy: a robust intervention. *Sex Transm Dis* 2010,**37**:602-607.
69. Young T, de Kock A, Jones H, Altini L, Ferguson T, van de Wijgert J. A comparison of two methods of partner notification for sexually transmitted infections in South Africa: patient-delivered partner medication and patient-based partner referral. *Int J STD AIDS* 2007,**18**:338-340.
70. Workowski KA, Berman S, Centers for Disease C, Prevention. Sexually transmitted diseases treatment guidelines, 2010. *MMWR Recomm Rep* 2010,**59**:1-110.
71. Department of Health RoSA. National consolidated guidelines for the prevention of mother-to-child transmission of HIV (PMTCT) and the management of HIV in children, adolescents and adults. In: http://www.sahivsoc.org/upload/documents/HIV%20guidelines%20_Jan%202015.pdf ; 2014.
72. Archary D, Liebenberg LJ, Werner L, Tulsi S, Majola N, Naicker N*, et al.* Randomized Cross-Sectional Study to Compare HIV-1 Specific Antibody and Cytokine Concentrations in Female Genital Secretions Obtained by Menstrual Cup and Cervicovaginal Lavage. *PLoS One* 2015,**10**:e0131906.
73. Liquet B, Le Cao KA, Hocini H, Thiebaut R. A novel approach for biomarker selection and the integration of repeated measures experiments from two assays. *BMC Bioinformatics* 2012,**13**:325.
